# Isopeptide bond formation mediated by δ-selenolysine for chemical ubiquitination

**DOI:** 10.3389/fchem.2023.1307254

**Published:** 2023-11-21

**Authors:** Tatsunari Akiyama, Yusuke Tanaka, Ryo Okamoto, Yasuhiro Kajihara, Masayuki Izumi

**Affiliations:** ^1^ Department of Chemistry and Biotechnology, Faculty of Science and Technology, Kochi University, Kochi, Japan; ^2^ Department of Chemistry, Graduate School of Science, Osaka University, Osaka, Japan; ^3^ Forefront Research Center, Graduate School of Science, Osaka University, Osaka, Japan

**Keywords:** ubiquitin, δ-selenolysine, enzymatic optical resolution, isopeptide bond, peptide ligation, deselenization

## Abstract

Protein ubiquitination is involved in nearly all biological processes in Eukaryotes. To gain precise insights into the function of ubiquitination in these processes, researchers frequently employ ubiquitinated protein probes with well-defined structures. While chemical protein synthesis has afforded a variety of ubiquitinated protein probes, there remains a demand for efficient synthesis methods for complex probes, such as ubiquitinated glycoproteins and ubiquitinated cysteine-containing proteins. In this study, we introduce a new method to obtain ubiquitinated proteins through isopeptide bond formation mediated by δ-selenolysine residues. We synthesized δ-selenolysine derivatives in both L- and D-forms starting from DL-δ-hydroxy-DL-lysine, accomplished by substituting the δ-mesylate with KSeCN and by enzymatic optical resolution with L- and D-aminoacylase. We synthesized ubiquitin (46–76)-α-hydrazide with a δ-seleno-L-lysine residue at position 48, as well as ubiquitin (46–76)-α-thioester, using solid-phase peptide synthesis. Subsequently, the δ-selenolysine-mediated ligation of these peptides, followed by one-pot deselenization, provided the desired isopeptide-linked ubiquitin peptide. The new δ-selenolysine-mediated isopeptide bond formation offers an alternative method to obtain complex ubiquitin- and ubiquitin-like probes with multiple post-translational modifications. These probes hold promise for advancing our understanding of ubiquitin biology.

## 1 Introduction

Protein ubiquitination is a major post-translational modification in Eukaryotes and is involved in nearly all biological processes, i.e., protein degradation, DNA repair, and cell signaling. Hence, unraveling its precise role in health and disease is important (Chaugule and Walden, 2016; Swatek and Komander, 2016). Ubiquitin (Ub) is a 76-mer protein found in almost all Eukaryotes. In protein ubiquitination, the C-terminal carboxylate of Ub is attached to the ε-amino group of the lysine residue in the acceptor protein through an isopeptide bond. Ub can form homopolymers, heteropolymers, and branched polymers through its seven Lys residues and one N-terminal Met. This structural diversity allows the formation of the “Ub code” (Komander and Rape, 2012; Swatek and Komander, 2016).

To gain precise insights into the complex Ub codes formed by diverse polyUb chains, researchers frequently employ chemical probes with polyUb chains having a defined linkage attached to a defined position of the acceptor protein (Mulder et al., 2020). Certain polyUb chains with defined linkage and length can be prepared using specific E2 or E3 enzymes. However, this method has some limitations, e.g., polyUb chains attach to the acceptor protein (Faggiano et al., 2016). Chemical protein synthesis has been extensively developed to overcome these limitations (Mali et al., 2017; Gui et al., 2021; Huppelschoten and van der Heden van Noort, 2022). Three strategies have been developed for the chemical synthesis of native isopeptide bonds: Auxiliary mediated ligation, first reported by Muir (Chatterjee et al., 2007); δ-mercaptolysine- (Kumar et al., 2009) or γ-mercaptolysine- (Yang et al., 2009; Merkx et al., 2013) mediated ligation; and isoUb ligation (Tang et al., 2017).

δ-Mercaptolysine-mediated ligation has been extensively used for the synthesis of diUb chains (Kumar et al., 2010), tetra-ubiquitinated peptides (Bavikar et al., 2012), polyubiquitinated α-synuclein (Haj-Yahya et al., 2013), and ubiquitinated and glycosylated H2B (Seenaiah et al., 2015). We also used δ-mercaptolysine-mediated ligation for the synthesis of ubiquitinated glycoprotein CCL1 in different folding states (Izumi et al., 2020). These reports demonstrate the usefulness of δ-mercaptolysine-mediated ligation for the synthesis of complex ubiquitinated protein probes. However, this approach requires subsequent desulfurization after isopeptide bond formation, necessitating the orthogonal protection of cysteine side-chain thiols to prevent undesired desulfurization. Unfortunately, the efficiency and overall yield of this process are compromised by additional deprotection and HPLC purification steps.

Metanis reported that deselenization with tris(2-carboxyethyl)phosphine (TCEP) proceeds selectively in the presence of free cysteine thiol without concomitant desulfurization (Metanis et al., 2010; Reddy et al., 2016). To avoid the risk of desulfurizing native Cys residues, γ-selenolysine-mediated ligation was developed (Dardashti et al., 2020). In the same study, they also attempted the synthesis of a δ-selenolysine derivative from L-glutamic acid. However, they could not obtain the desired product with good yields and purity. Here, we report the synthesis of δ-selenolysine derivative in both L- and D-forms and its use in the formation of traceless isopeptide bonds. We sought to prepare δ-selenolysine in both L- and D-forms because doing so will allow access to the ubiquitinated protein in both its L- and D-forms (Lander et al., 2023), which is required for racemic protein crystallography (Yeates and Kent, 2012; Yan et al., 2017). Although the quasi-racemic and monomer/oligomer quasi-racemic X-ray structures of diUb and triUb were reported (Pan et al., 2016; Wang et al., 2016), true racemic protein crystallography will be more useful for elucidating the X-ray structures of ubiquitinated proteins.

## 2 Results and discussion

### 2.1 Synthesis of δ-selenolysine derivatives L-9 and D-9

To synthesize the δ-seleno-L-lysine derivative suitably protected for solid-phase peptide synthesis, we turned our attention to the synthesis route of δ-mercapto-L-lysine derivatives. Kumar et al. reported the synthesis starting from L-glutamic acid (Kumar et al., 2009; Haj-Yahya et al., 2010), while Virdee et al. used δ-hydroxy-L-lysine as a starting material (Virdee et al., 2011). We decided to examine whether commercially available racemic DL-δ-hydroxy-DL-lysine can be employed to synthesize the δ-selenolysine derivative in both its L- and D-form after optical resolution.

**SCHEME 1:**
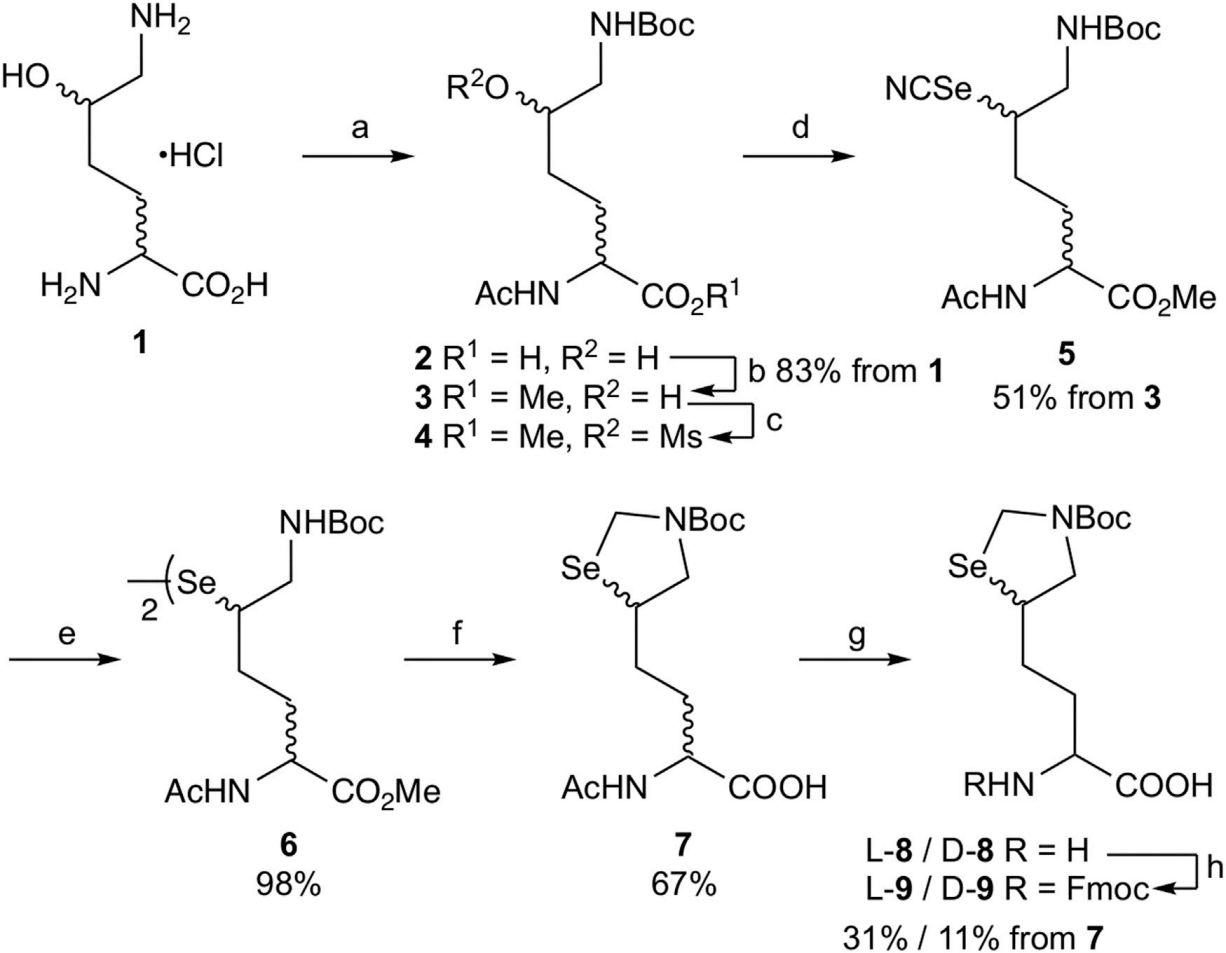
Synthesis of δ-selenolysine derivatives **L-9** and **D-9**. Reagents and conditions: (a) i. NaHCO_3_/CuSO_4_·5H_2_O, then Boc_2_O, ii. 8-quinolinol, iii. Ac_2_O/NaOH/1,4-dioxane:H_2_O = 1:1; (b) K_2_CO_3_/MeI/DMF; (c) MsCl/DIEA/DCM; (d) KSeCN/MeCN/60°C; (e) NaBH_4_/95% EtOH/THF/0°C; (f) i. TFA/DCM, ii. NaBH_4_/EtOH then 37% aq. HCHO, iii. Boc_2_O/K_2_CO_3_/water/1.4-dioxane, iv. NaOH/aq. THF; (g) L-aminoacylase (or D**-**aminoacylase)/0.2 M phosphate buffer pH 8.0/37°C; (h) Fmoc-OSu/THF.

To employ L- or D-aminoacylase for enzymatic optical resolution (Morishita et al., 2015), the α-amino group should be protected with an acetyl group (Scheme 1). Selective protection of the ε-amino group of DL-δ-hydroxy-DL-lysine hydrochloride **1** was achieved by forming a copper (II) complex and then reacting it with Boc_2_O (Virdee et al., 2011). Acetylation of the α-amino group with acetic anhydride and sodium hydroxide yielded acetamide **2**, which was subsequently treated with iodomethane for 3.5 h under basic conditions to obtain methyl ester **3** in 83% yield from **1**. Prolonging this reaction (5 h) afforded δ-lactone as a byproduct along with methyl ester **3**. In such a case, δ-lactone can be easily converted back to **3** by treating the resulting mixture with sodium methoxide. Next, δ-alcohol **3** was treated with MsCl to produce δ-mesylate **4** for the substitution reaction. Reacting **4** with potassium selenocyanate in acetonitrile at 60°C for 42 h yielded δ-selenocyanate **5** in 51% yield in two steps (Malins and Payne, 2012). Selenocyanate **5** was converted to diselenide **6** (98% yield) by treatment with NaBH_4_. Then, diselenide **6** was converted to carboxylate **7** by the following reaction sequence: the Boc group was removed by treatment with TFA. The diselenide bond was reduced by NaBH_4_, and the resulting 1,2-aminoselenol group was protected as selenazolidine by treatment with 37% HCHO under acidic pH, and the ε-imino group was protected again with the Boc group to obtain methyl ester. However, undesired partial hydrolysis of methyl ester was observed during this reaction sequence, and the isolation of methyl ester reduced the overall yield. Thus, the crude product was treated with aqueous NaOH, and carboxylate **7** was obtained in 67% yield from **6** after purification via silica gel column chromatography.

**FIGURE 1:**
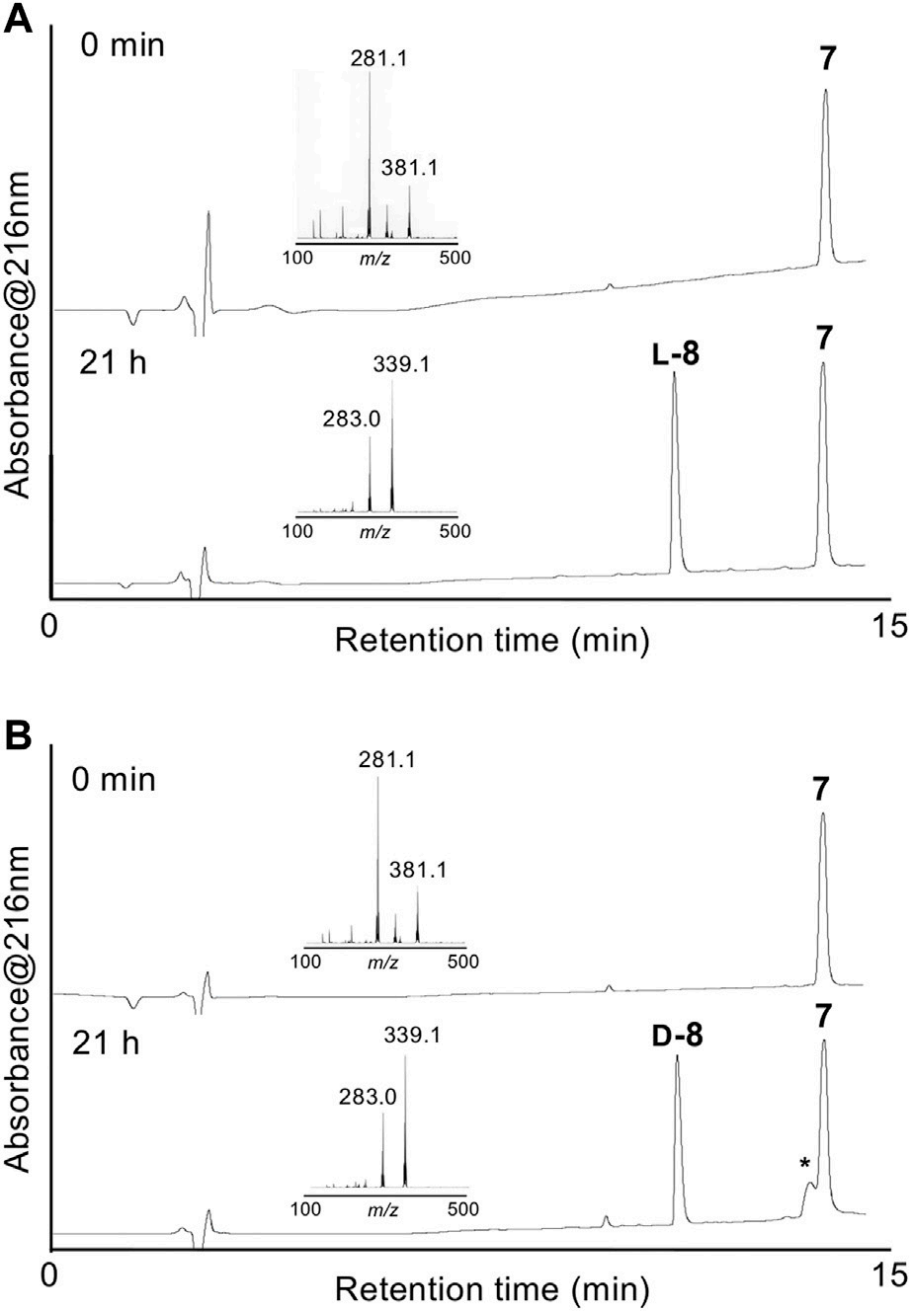
HPLC chromatograms of enzymatic optical resolution with L-aminoacylase **(A)** and D-aminoacylase **(B)** at 0 min and 21 h. Insets show the LC/MS spectra of starting **7** and **L-8** or **D-8**. *Peak associated with the commercial D-aminoacylase sample.

Enzymatic optical resolution using L-aminoacylase was examined using racemic DL-δ-seleno-DL-lysine **7**. Racemic **7** was treated with L-aminoacylase in phosphate buffer at pH 8, and the reaction progress was monitored by LC/MS. As shown in Figure 1A, a new peak corresponding to the desired deacetylated compound **L-8** was observed. However, the peak of **7** did not disappear after 21 h. Fmoc-OSu was added to the reaction mixture to produce Fmoc-protected **L-9** in 31% isolated yield from racemic **7**. We also examined the enzymatic optical resolution of racemic **7** by D-aminoacylase. This reaction also resulted in two peaks: one corresponding to the deacetylated product **D-8** and the other to remaining **7** (Figure 1B). Treatment with Fmoc-OSu produced **D-9** in 11% isolated yield from racemic **7**. We speculate that the small-scale reaction resulted in a lower yield of **D-9** than that of **L-9**, because the LC/MS analysis results suggested that the enzymatic deacetylation of **7** by L-aminoacylase and D-aminoacylase proceeded to a similar degree. The **7** remaining after hydrolysis with D-aminoacylase was isolated and treated with L-aminoacylase. The HPLC peak corresponding to **7** weakened, while the **L-8** peak appeared. However, the peak corresponding to **7** did not disappear even after 18 h (Supplementary Figure S1A). The results suggest that enzymatic hydrolysis did not proceed to completion. Compound **7** was also not completely hydrolyzed by 24-h treatment with L-aminoacylase followed by additional 24-h treatment with D-aminoacylase (Supplementary Figure S1B). These results suggest that the remaining **7** after each enzymatic hydrolysis is not optically pure. Thus, an efficient approach for obtaining both **L-9** and **D-9** could involve recovering the unreacted **7** from the L-aminoacylase reaction mixture and utilizing it as a substrate for D-aminoacylase.

To determine the absolute configuration at the α-position of synthetic **L-9** and **D-9**, pentapeptide FKPSD was synthesized by Fmoc-solid-phase peptide synthesis (SPPS) using both **L-9** and **D-9** (Supplementary Scheme S1) (Kumar et al., 2009). Pentapeptides containing a δ-selenolysine residue **L-10** and **D-10** were treated with MeONH_2_·HCl to convert them to diselenides **L-11** and **D-11**, which were then deselenized by treatment with TCEP to yield pentapeptides **L-12** and **D-12**, respectively. We also synthesized pentapeptides F(L-K)PSD and F(D-K)PSD using commercial Fmoc-L-Lys(Boc) and Fmoc-D-Lys(Boc), respectively, to compare them with **L-12** and **D-12**. The retention time of each pentapeptide on reversed-phase (RP) HPLC confirmed that **L-12** is F(L-K)PSD and **D-12** is F(D-K)PSD (Supplementary Figure S2). HPLC analyses did not show any F(D-K)PSD or F(L-K)PSD peak from the pentapeptide sample synthesized using **L-9** or **D-9,** respectively. Hence, it can be suggested that the optical purity of the α-position of **L-9** and **D-9** is high enough to be used in the SPPS (Supplementary Figure S2).

### 2.2 δ-Selenolysine-mediated isopeptide bond formation

**SCHEME 2:**
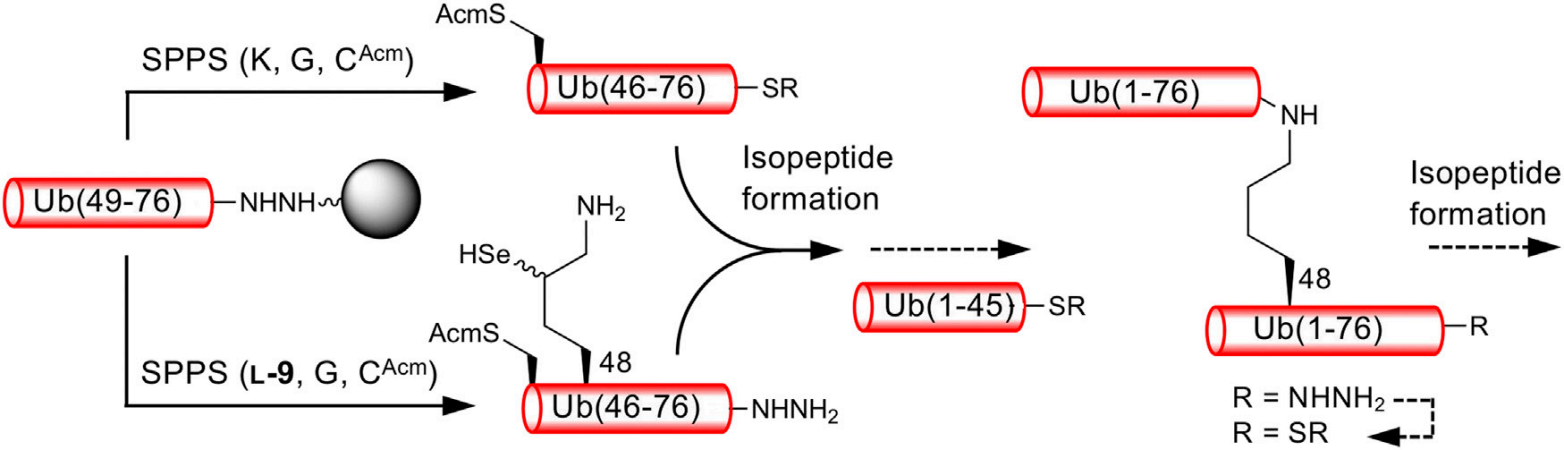
Synthetic strategy for K48-linked diubiquitinated protein probes. Reactions reported here are presented by solid arrows.

We next examined the formation of isopeptide bonds using Ub (46–76) peptide fragment and ligation position at Lys48. We selected Lys48 of Ub as a linkage position because protein degradation through the ubiquitin-proteasome system mediated by Lys48-linked polyUb is well-studied (Hershko and Ciechanover, 1998). In addition, the Ub (49–76) peptide resin can be used as a common intermediate for the synthesis of both proximal and distal Ub (46–76) fragments, Ub (46C^Acm^–48SeK–76) and Ub (46C^Acm^–76), respectively (Scheme 2). The use of the Ub (46–76) peptide fragment with the mutation of Ala46 to Cys allows the fabrication of a full-length Ub polypeptide by native chemical ligation–desulfurization with Ub (1–45)-α-thioester (Erlich et al., 2010). Side-chain thiol of the N-terminal Cys was protected with an acetamidomethyl (Acm) group. Although SPPS of a full-length Ub chain is already reported (El Oualid et al., 2010; Bavikar et al., 2012), synthesizing such chains containing δ-selenolysine in their middle does not seem practical. We synthesized both the distal and proximal Ub (46–76) peptide fragments as peptide hydrazide, because, after isopeptide bond formation, the C-terminal hydrazide of the isopeptide can be converted to thioester and further used for the chemical polyubiquitination of the acceptor protein (Scheme 2).

Ub (46C^Acm^–76)-α-hydrazide was synthesized using Fmoc-SPPS on the Fmoc-hydrazine 2-chlorotrityl resin (Huang et al., 2014). To synthesize the 31-mer peptide on the polystyrene-based 2-chlorotrityl resin, the loading of the first amino acid was adjusted to 0.4–0.5 mmol/g and the coupling of the amino acid was carried out using Fmoc-AA (10 equiv) with DIC/OxymaPure in NMP at 60°C for 20 min (Bacsa et al., 2008). Dipeptides Fmoc-Leu-Ser(psiMe, Mepro) and Fmoc-Asp(OtBu)-(Dmb)Gly were used at positions 56–57 and 52–53, respectively, according to the assembly of a full-length Ub chain. Boc-Cys(Acm) was coupled at the N-terminus. Global deprotection and cleavage by treatment with TFA/TIPS/water (95/2.5/2.5) yielded the desired Ub (46C^Acm^–76)-α-hydrazide **13** in 17% yield after RP-HPLC purification using a C4 column (Supplementary Figure S3). Then, the peptide-hydrazide **13** was converted to thioester **14** in 70% yield by treatment with acetylacetone and 4-mercaptophenylacetic acid (MPAA) at pH 3 (Supplementary Figure S4) (Flood et al., 2018). The Ub (46–76) peptide fragment containing a δ-seleno-L-lysine residue at position 48 was synthesized by a similar procedure. Coupling of **L-9** (2 equiv) with the Ub (49–76)-peptide resin was performed with DIC/OxymaPure at 60°C for 20 min. The coupling was performed twice to ensure the incorporation of **L-9** into the resin-bound peptide. After the completion of peptide elongation, global deprotection and cleavage were performed using TFA/TIPS/water/MeONH_2_·HCl (94/2.5/2.5/0.2) (Dardashti et al., 2020). The desired Ub (46C^Acm^–48K^Sez^–76)-α-hydrazide **15** was obtained in 10% yield after RP-HPLC purification (Supplementary Figure S5). We also attempted to synthesize Ub (46C–48K^Sez^–76)-α-hydrazide with an N-terminal free cysteine residue, which can be first ligated with Ub (1–45)-α-thioester and then used for δ-selenolysine-mediated isopeptide bond formation. However, a selenosulfide bond was formed between Cys46 and δ-selenoLys48 residues, suggesting that selenazolidine is unstable at neutral pH when a free thiol group is present in the same peptide. Protecting selenols in δ-selenolysine with stable protecting groups such as methoxybenzyl might be helpful in avoiding undesired deprotection.

**FIGURE 2:**
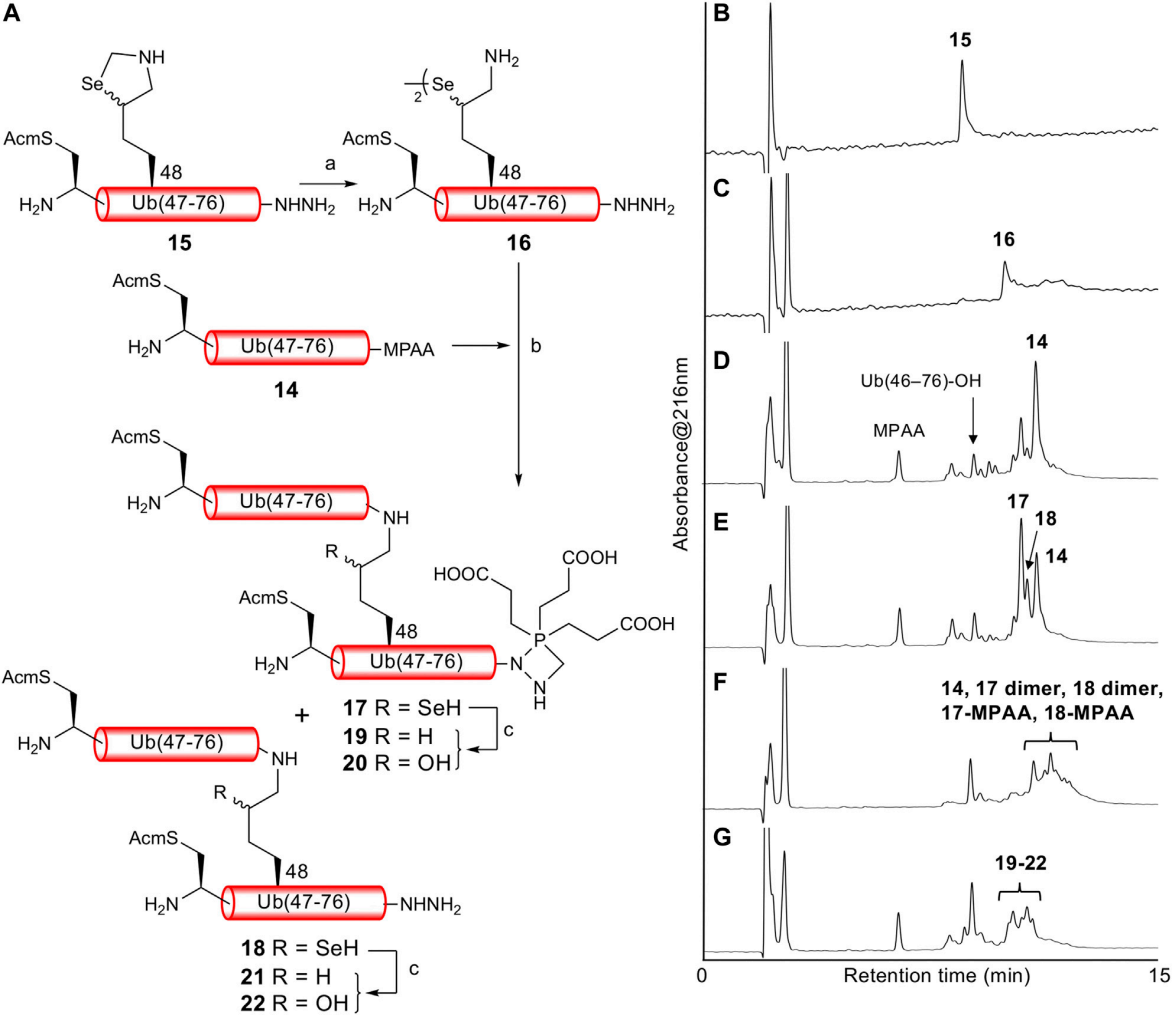
**(A)** First attempt of one-pot δ-selenolysine-mediated isopeptide bond formation. Reagents and conditions: (a) CuCl_2_/6 M Gn·HCl/0.2 M phosphate buffer, pH 6; (b) TCEP (5 equiv)/6 M Gn·HCl/0.2 M phosphate buffer, pH 6.5; (c) TCEP (100 equiv)/6 M Gn·HCl/0.2 M phosphate buffer, pH 5. Time course of analytical HPLC chromatograms. **(B)** Selenazolidine deprotection at 1 min. **(C)** Selenazolidine deprotection at 4.5 h. **(D)** Ligation at 20 min. **(E)** Ligation at 1.5 h. **(F)** Ligation at 13 h. **(G)** Deselenization at 16 h.

**FIGURE 3:**
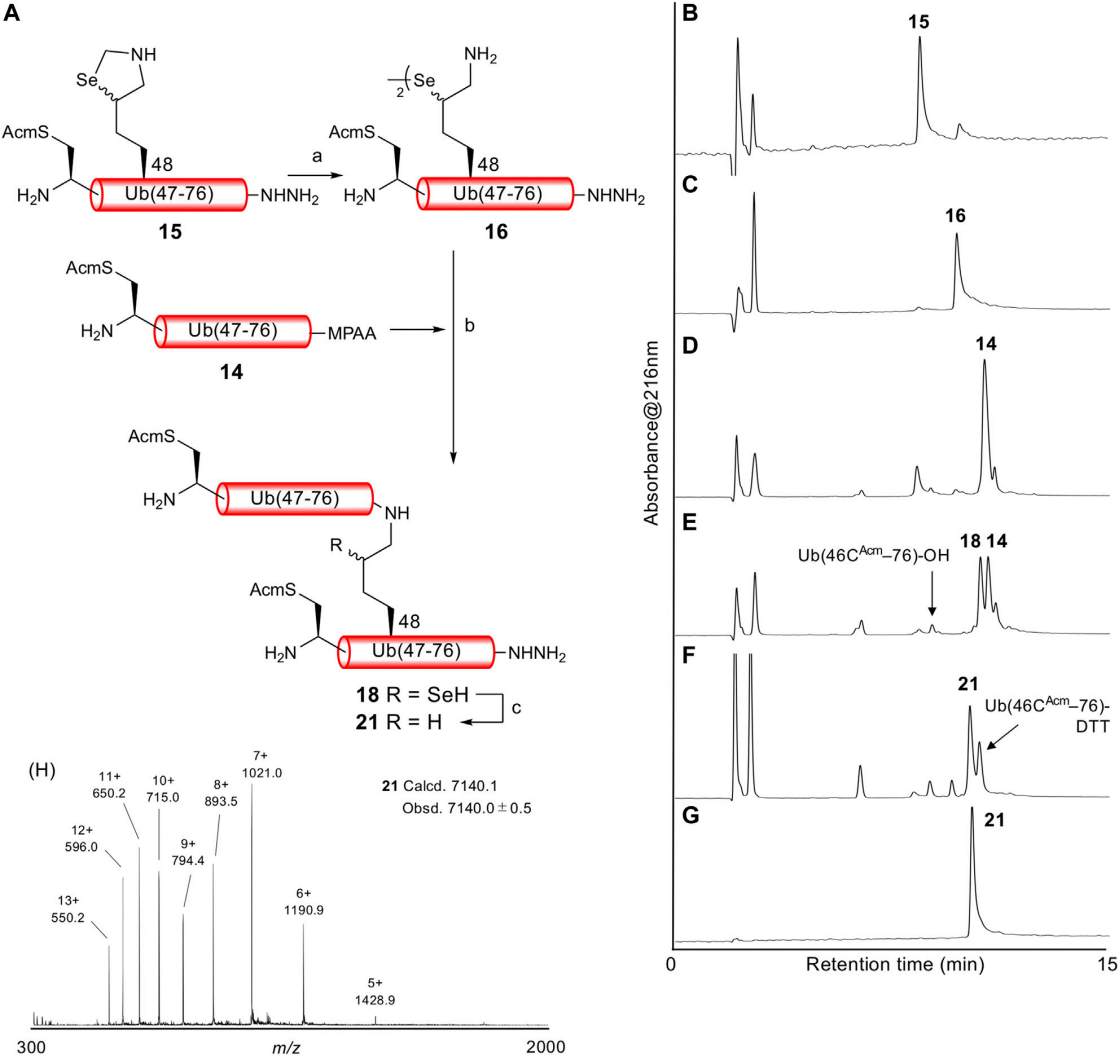
**(A)** One-pot δ-selenolysine-mediated isopeptide bond formation. Reagents and conditions: (a) CuCl_2_ (1.5 equiv)/MeONH_2_·HCl (5 equiv)/6 M Gn·HCl/0.2 M phosphate buffer, pH 6; (b) TCEP (5 equiv)/6 M Gn·HCl/0.2 M phosphate buffer, pH 6; (c) TCEP (50 equiv)/DTT (50 equiv)/6 M Gn·HCl/0.2 M phosphate buffer, pH 5. Time course of analytical HPLC chromatograms. **(B)** Selenazolidine deprotection at 1 min. **(C)** Selenazolidine deprotection at 30 min. **(D)** Ligation at 1 min. **(E)** Ligation at 2 h. **(F)** Deselenization at 13 h. Peak eluted after **21** is Ub(46C^Acm^–76)-DTT produced from the remaining **14**. **(G)** Purified **21**. **(H)** ESI-MS spectrum of purified **21**.

With both Ub (46C^Acm^–76)-α-thioester **14** and Ub (46C^Acm^–48K^Sez^–76)-α-hydrazide **15** in hand, we examined the δ-selenolysine-mediated isopeptide bond formation. We investigated the one-pot copper-mediated selenazolidine deprotection and native chemical ligation conditions (Zhao and Metanis, 2019). In our first attempt (Figure 2; Supplementary Figure S6), we initially treated selenazolidine **15** with CuCl_2_ at pH 6 to produce diselenide **16** and then added thioester **14** and TCEP to the solution. The desired ligated product **18** was observed after 1.5 h, along with **17** (+262 Da) as a major byproduct. We subsequently added TCEP and adjusted the pH to 5 for deselenization. The LC/MS analysis results showed the formation of the desired deselenized product **21**; however, deselenized **19** (+262 Da) was still a major byproduct. Oxidized byproducts **20** and **22** were also formed because of insufficient degassing of the reaction buffer. We speculated that the major byproduct with a molecular weight of +262 Da was formed by a reaction with formaldehyde released from selenazolidine and TCEP with a C-terminal hydrazide. To confirm this speculation, selenazolidine **15** was deprotected by treatment with CuCl_2_ and the liberated formaldehyde was removed by solid-phase extraction. Then, diselenide **16** was ligated with thioester **14**. This stepwise synthesis did not yield any byproduct of molecular weight +262 Da, suggesting that formaldehyde was involved in the formation of this byproduct (data not shown). Finally, we performed one-pot selenazolidine deprotection–δ-selenolysine-mediated ligation–deselenization (Figure 3; Supplementary Figure S7). First, selenazolidine **15** was treated with CuCl_2_ for 30 min in the presence of MeONH_2_·HCl as a scavenger of formaldehyde to yield diselenide **16**. Then, thioester **14** and 5 equiv of TCEP were added to perform ligation for 2 h. Deselenization was performed by adding 50 equiv of both TCEP and DTT at pH 5 for 13 h (Wang et al., 2020). The desired isopeptide Ub [46C^Acm^–48K{Ub (46C^Acm^–76)}–76)]-α-hydrazide **21** was obtained in 53% yield and in good purity after RP-HPLC purification. The structure of **21** was confirmed by trypsin digestion (Supplementary Figure S8). The LC/MS analysis results confirmed the formation of the desired isopeptide fragment. Hence, it can be suggested that the formation of isopeptide bonds involves a δ-selenolysine residue at position 48.

The reaction time of δ-selenolysine-mediated ligation (2 h) was much shorter than that of the γ-selenolysine-mediated ligation (22 h) (Dardashti et al., 2020). However, these two reactions cannot be compared directly because the sequence of peptides containing γ/δ-selenolysine residues and peptide thioesters used are different, although both have Gly–Gly at the C-terminus. Additionally, we used an excess amount of highly reactive MPAA-thioester to push the reaction to completion in a short period of time because, in the selenium-mediated ligation, a longer reaction time results in the formation of inactive diselenide, which slows down the reaction. We assume that the faster reaction of δ-selenolysine-mediated ligation is not because of the five-membered ring transition rather than the six-membered ring transition because γ- and δ-mercaptolysine-mediated ligation proceeds at a similar rate (Merkx et al., 2013).

## 3 Materials and methods

For general experimental and instrumental methods, and full compound characterization, see the Supplementary Material S1.

### 3.1 *N*-α-acetyl-*N*-ε-(*tert*-butoxycarbonyl)-δ-DL-hydroxy-DL-lysine methyl ester (3)

Crude *N*-ε-(*tert*-butoxycarbonyl)-δ-DL-hydroxy-DL-lysine (2.64 g), prepared from δ-DL-hydroxy-DL-lysine hydrochloride **1** (2.00 g, 10.1 mmol) by Chin’s procedure (Virdee et al., 2011), was dissolved in a mixture of water and 1,4-dioxane (1:1, v/v, 13.5 mL). Ac_2_O (1.9 mL, 20.1 mmol) was divided into three portions and added to the solution. The pH of the resulting solution was adjusted each time to 11 with 5 M NaOH. After adding all Ac_2_O, the solution was carefully neutralized with 1 M HCl and concentrated *in vacuo*. The residue was desalted by chromatography on C18 silica gel (washed with water and then eluted with 15% aqueous MeCN) to yield *N*-α-acetyl-*N*-ε-(*tert*-butoxycarbonyl)-δ-DL-hydroxy-DL-lysine **2** (3.52 g) as a colorless syrup.; HR-ESI-FTMS *m/z* calcd for C_13_H_24_N_2_O_6_ [M + Na]^+^ 327.1527, found: 327.1527; ^1^H NMR (400 MHz, CD_3_OD) δ 4.30–4.21 (m, 1H), 3.67–3.53 (m, 1H), 3.17–3.07 (m, 1H), 3.07–2.94 (m, 1H), 2.00 (s, 3H), 1.96–1.50 (m, 4H), 1.45 (s, 9H); ^13^C NMR (100 MHz, CD_3_OD) δ 177.8, 177.7, 171.12, 171.08, 157.2, 78.6, 70.3, 69.8, 54.9, 54.5, 46.0, 30.6, 30.4, 28.8, 28.7, 27.4, 21.4.

To a solution of **2** (3.52 g) in DMF (100 mL), K_2_CO_3_ (3.22 g, 23.3 mmol) was added, and the mixture was stirred for 10 min at room temperature. Iodomethane (1.1 mL, 17.5 mmol) was added to the suspension, and the resulting suspension was stirred for 3.5 h at room temperature. The solid was filtered off, and the filtrate was concentrated *in vacuo*. The residue was concentrated again from toluene. The residue was dissolved in EtOAc and washed with brine. The organic layer was dried over MgSO_4_, filtered, and concentrated. The residue was purified by silica gel chromatography (EtOAc) to yield **3** (2.65 g, 83% from **1**) as a colorless syrup.; HR-ESI-FTMS *m/z* calcd for C_14_H_26_N_2_O_6_ [M + Na]^+^ 341.1683, found: 341.1684; ^1^H NMR (400 MHz, CDCl_3_) δ 6.43, 6.36 (br, 1H), 5.00 (br, 1H), 4.66–4.58 (m, 1H), 3.75 (s, 3H), 3.71 (br, 1H), 3.31–3.21 (br, 1H), 3.09–2.97 (m, 1H), 2.03 (s, 3H), 2.00–1.75 (m, 2H), 1.57–1.43 (m, 1H), 1.45 (s, 9H); ^13^C NMR (100 MHz) δ 172.93, 172.89, 170.3, 170.1, 157.1, 156.9, 79.9, 79.7, 71.2, 71.0, 52.50, 52.45, 52.0, 51.8, 46.7, 46.6, 30.1, 30.0, 29.3, 28.6, 28.4, 23.16, 23.13.

### 3.2 *N*-α-acetyl-*N*-ε-(*tert*-butoxycarbonyl)-δ-DL-cyanoseleno-DL-lysine methyl ester (5)

A solution of **3** (2.23 g, 7.00 mmol) in DCM (30 mL) and DIEA (2.4 mL, 14 mmol) was cooled in an ice bath, and MsCl (0.651 mL, 8.4 mmol) was added dropwise. The solution was gradually warmed to room temperature and stirred for 2 h. The solution was diluted with DCM and washed with saturated aqueous NH_4_Cl and brine, sequentially. The organic layer was dried over MgSO_4_, filtered, and concentrated *in vacuo* to yield *N*-α-acetyl-*N*-ε-(*tert*-butoxycarbonyl)-δ-DL-methanesulfornyloxy-DL-lysine methyl ester **4** (2.90 g) as a syrup.; ESI MS calcd. for C_15_H_28_N_2_O_8_S [M + Na]^+^ 419.1, found 419.1; ^1^H NMR (400 MHz, CDCl_3_) δ 6.21 (br, 1H), 4.96 (br, 1H), 4.78–4.72 (m, 1H), 4.64–4.56 (m, 1H), 3.76 (s, 3H), 3.47–3.43 (m, 1H), 3.32–3.26 (m, 1H), 3.06 (s, 3H), 2.04 (s, 3H), 2.01–1.97 (m, 1H), 1.87–1.67 (m, 3H), 1.45 (s, 9H); ^13^C NMR (100 MHz) δ 172.4, 170.2, 156.1, 80.9, 80.1, 52.6, 51.8, 51.6, 43.8, 43.6, 38.5, 28.3, 28.22, 28.18, 28.0, 27.7, 23.1.

To a solution of **4** (2.90 g) in MeCN (40 mL), KSeCN (2.11 g, 14.6 mmol) was added, and the mixture was stirred for 42 h at 60°C. The solution was concentrated, and the residue was dissolved in DCM (15 mL) and poured onto water (30 mL). The aqueous layer was extracted three times with DCM. The combined organic layers were dried over MgSO_4_, filtered, and concentrated *in vacuo*. The residue was purified by silica gel chromatography (EtOAc:hexane = 2:1 then EtOAc) to yield **5** (1.45 g, 51%) as a syrup. HR-ESI-TOFMS *m/z* calcd for C_15_H_25_N_3_O_5_Se [M + Na]^+^ 430.0852, found 430.0868; ^1^H NMR (400 MHz, CDCl_3_) δ 6.65 (s, 1H), 5.54 (s, 1H), 4.70–4.56 (m, 1H), 3.77 (s, 3H), 3.65–3.48 (m, 2H), 3.43–3.30 (m, 1H), 2.18–2.05 (m, 1H), 2.04 (s, 3H), 2.00–1.68 (m, 3H), 1.45 (s, 9H); ^13^C NMR (100 MHz, CDCl_3_) δ 172.4 (Ac), 170.3 (COOH), 156.1, 156.0 (Boc-CO), 100.8, 100.6 (SeCN), 80.2 (tBu-C), 52.7, 52.6 (OMe), 51.6, 51.4 (α), 49.9, 49.8 (δ), 45.6 45.1 (ε), 30.3, 29.5, 28.3 (β,γ), 23.0 (tBu).

### 3.3 *N*-α-acetyl-*N*-ε-(*tert*-butoxycarbonyl)-δ-DL-seleno-DL-lysine methyl ester dimer (6)

A solution of NaBH_4_ (18 mg, 0.47 mmol) in 95% aqueous EtOH (0.5 mL) was added dropwise to a solution of **5** (94.4 mg, 0.23 mmol) in THF (3.5 mL) at 0°C. The solution was stirred for 15 min at 0°C, diluted with EtOAc (10 mL), and poured onto 1 M aqueous HCl (15 mL). The organic layer was separated, and the aqueous layer was extracted with EtOAc (3 × 10 mL). Combined organic layers were dried over MgSO_4_, filtered, and concentrated. The residue was purified by silica gel chromatography (EtOAc) to yield **6** (86.6 mg, 98%) as a yellow oil.; ESI MS calcd. for C_28_H_50_N_4_O_10_Se_2_ [M + Na]^+^ 785.2, found: 785.2; ^1^H NMR (400 MHz, CDCl_3_) δ 6.77–6.59 (m, 1H), 5.45–5.10 (m, 1H), 4.61–4.48 (m, 1H), 3.72 (s, 3H), 3.38–3.22 (m, 2H), 3.09–2.96 (m, 1H), 2.12–2.05 (m, 1H), 2.03 (s, 3H), 1.97–1.52 (m, 3H), 1.45 (s, 9H); ^13^C NMR (100 MHz) δ 172.73, 172.67, 170.4, 170.2, 156.2, 79.6, 52.5, 51.82, 51.78, 45.8, 45.7, 45.2, 45.0, 44.8, 30.4, 29.7, 29.1, 29.0, 28.4, 23.0.

### 3.4 *N*-α-acetyl-*N*-ε-(*tert*-butoxycarbonyl)-δ,ε-*Se,N*-methylene-δ-DL-seleno-DL-lysine (7)

To a solution of **6** (0.226 g, 0.297 mmol) in DCM (4 mL), 10% v/v TFA/DCM (0.45 mL, 5.9 mmol) was added, and the mixture was stirred for 3 h at room temperature. The solution was concentrated *in vacuo*, and the residue was concentrated twice from toluene to yield crude *N*-α-acetyl-δ-DL-seleno-DL-lysine methyl ester dimer (0.307 g) as a syrup. The syrup was dissolved in 0.05 M aqueous NaOH (5.9 mL) and EtOH (1.6 mL). The solution was bubbled with Ar for 5 min, and NaBH_4_ (39.3 mg, 1.01 mmol) was added in three portions to this solution over 10 min under an Ar atmosphere. When the solution was stirred for 30 min, it became colorless. It was then cooled to 0°C, and the pH was adjusted to 4–5 by adding 5 M HCl. To this solution, 37% aqueous formaldehyde (222 μL, 3.0 mmol) was added over 25 min. The resulting solution was stirred first for 30 min at 0°C and then for 20 h at room temperature. The solution was concentrated *in vacuo* at 30°C, and the resulting aqueous solution was lyophilized to yield crude *N*-α-acetyl-δ,ε-*Se,N*-methylene-δ-DL-seleno-DL-lysine methyl ester (0.460 g) as a white solid. The solid was dissolved in water (3.1 mL), and K_2_CO_3_ (94.2 mg, 0.888 mmol) was added to it at 0°C. The resulting solution was stirred for 10 min at 0°C, and then a solution of Boc_2_O (0.163 mL, 0.71 mmol) in 1,4-dioxane (3.1 mL) was added dropwise. The obtained solution was first stirred for 5 min at 0°C, and then for an additional 14 h at room temperature. The solution was concentrated to yield crude *N*-α-acetyl-*N*-ε-(*tert*-butoxycarbonyl)-δ,ε-*Se,N*-methylene-δ-DL-seleno-DL-lysine methyl ester (0.602 g).; ESI MS C_15_H_26_N_2_O_5_Se [M + H]^+^ calcd: 394.1, found: 394.4; ^1^H NMR (400 MHz, CDCl_3_) δ 6.29–6.02 (m, 1H), 4.85–4.72 (m, 1H), 4.68–4.51 (m, 2H), 3.76 (s, 3H), 3.75–3.68 (m, 2H), 3.66–3.55 (m, 1H), 2.03 (s, 3H), 1.99–1.50 (m, 4H), 1.48 (s, 9H); ^13^C NMR (100 MHz) δ 172.73, 172.67 (Ac), 170.4, 170.2 (COOH), 156.2 (Boc-CO), 79.6 (tBu-C), 52.5 (OMe), 51.82, 51.78 (α), 45.8, 45.7, 45.2, 45.0, 44.8 (δ,ε), 30.4, 29.7, 29.1, 29.0 (β,γ), 28.4 (tBu), 23.0 (Ac).

Crude methyl ester (0.602 g) was dissolved in THF (3 mL), and 1 M NaOH (3 mL) was added to the solution. The solution was stirred for 1.5 h at room temperature, and neutralized with Dowex 50W-X8 (H^+^ form). The resin was filtered off, and the filtrate was concentrated. The residue was purified by silica gel chromatography (EtOAc then EtOAc:MeOH = 4:1) to yield **7** (0.150 g, 67% from **6**) as a colorless syrup.; HR-ESI-TOFMS *m/z* calcd. for C_14_H_24_N_2_O_5_Se [M + Na]^+^ 403.0743, found: 403.0754; ^1^H NMR (400 MHz, CDCl_3_) δ 6.97–6.60 (br, 1H), 6.55–6.02 (br, 1H), 4.83–4.72 (m, 1H), 4.63–4.51 (m, 2H), 3.84–3.68 (m, 2H), 3.68–3.57 (m, 1H), 2.07 (s, 3H), 2.01–1.90 (m, 1H), 1.90–1.50 (m, 3H), 1.48 (s, 9H).

### 3.5 *N*-α-(9-fluorenylmethoxycarbonyl)-*N*-ε-(*tert*-butoxycarbonyl)-δ,ε-*Se,N*-methylene-δ-DL-seleno-L-lysine (L-9)

L-Aminoacylase (68 mg) was added to a solution of compound **7** (0.136 g, 0.36 mmol) in 0.2 M phosphate buffer (pH 8.0, 3.6 mL) and the resulting suspension was incubated for 21 h at 37°C. The LC/MS analysis [CAPCELLPAK C18 (2.0 mm × 150 mm), solvent A:solvent B = 80:20 to 5:95 over 10 min at a flow rate of 0.2 mL/min] results suggested that approximately half of **7** was converted to **L-8**.; ESI-MS *m/z* calcd. for C_12_H_22_N_2_O_4_Se [M + H]^+^ 339.1, found: 339.1; The suspension was centrifuged, and the supernatant was separated. The precipitate was washed twice with 0.2 M phosphate buffer. The combined supernatant and washings (total 5.6 mL) were added to a solution of Fmoc-OSu (0.0733 g, 0.217 mmol) in THF (5.6 mL). The solution was stirred overnight at room temperature. It was then concentrated *in vacuo* and the residue was purified by silica gel chromatography (EtOAc:hexane = 1:3, then 1:3 containing 1% AcOH) to yield **L-9** (0.0629 g, 31%) as a colorless syrup.; HR-ESI-FTMS *m/z* calcd. for C_27_H_32_N_2_O_6_Se [M + Na]^+^ 583.1318, found: 583.1317; ^1^H NMR (400 MHz, CDCl_3_) δ 7.75 (d, *J* 7.5 Hz, 2H), 7.63–7.50 (m, 2H), 7.39 (t, *J* 7.4 Hz, 2H), 7.30 (t, *J* 7.4 Hz, 2H), 5.66–5.34 (br, 1H), 4.86–4.65 (m, 1H), 4.63–4.49 (m, 1H), 4.48–4.34 (m, 2H), 4.21 (t, *J* 6.7 Hz, 1H), 3.90–3.57 (m, 3H), 2.08–1.93 (m, 1H), 1.93–1.53 (m, 3H), 1.48 (s, 9H); ^13^C NMR (100 MHz) δ 175.6 (COOH), 156.2, 154.3 (Fmoc-CO, Boc-CO), 143.8, 143.7, 141.3 (Fmoc-ArC), 127.8, 127.1, 125.1, 120.0 (Fmoc-ArCH), 81.2 (tBu-C), 67.2 (Fmoc-CH_2_O), 55.2 (br, ε), 53.4 (α), 47.1 (Fmoc-CH), 43.7 (br, δ), 39.7 (br, SeCH_2_N), 32.2, 31.6 (β,γ), 28.3 (tBu-Me).

### 3.6 *N*-α-(9-fluorenylmethoxycarbonyl)-*N*-ε-(*tert*-butoxycarbonyl)-δ,ε-*Se,N*-methylene-δ-DL-seleno-D-lysine (D-9)

D-Aminoacylase (7.0 mg) was added to a solution of compound **7** (14 mg, 37 μmol) in 0.2 M phosphate buffer (pH 8.0, 0.34 mL) and the resulting suspension was incubated for 21 h at 37°C. The LC/MS analysis results suggested that approximately half of **7** was converted to **D-8**.; ESI-MS *m/z* calcd. for C_12_H_22_N_2_O_4_Se [M + H]^+^ 339.1, found: 339.1.; A solution of Fmoc-OSu (7.6 mg, 23 μmol) in THF (0.59 mL) was added to this suspension. The resulting suspension was stirred overnight at room temperature and then concentrated *in vacuo*. The residue was purified by silica gel chromatography (EtOAc:hexane = 1:3, then 1:3 containing 1% AcOH) to yield **D-9** (2.3 mg, 11%) as a colorless syrup.; ESI-MS *m/z* calcd. for C_27_H_32_N_2_O_6_Se [M + Na]^+^ 583.1, found: 583.1; ^1^H NMR (500 MHz, CDCl_3_) δ 7.75 (d, *J* 7.5 Hz, 2H), 7.63–7.50 (m, 2H), 7.39 (t, *J* 7.4 Hz, 2H), 7.34 (t, *J* 7.4 Hz, 2H), 5.66–5.34 (br, 1H), 4.86–4.65 (m, 1H), 4.63–4.49 (m, 1H), 4.48–4.34 (m, 2H), 4.21 (t, *J* 6.7 Hz, 1H), 3.90–3.57 (m, 3H), 2.08–1.93 (m, 1H), 1.93–1.53 (m, 3H), 1.48 (s, 9H).

### 3.7 Determination of the absolute configuration of the α-position of L-9 and D-9

Aminomethyl-ChemMatrix resin was treated with HMPA (2.5 equiv), TBTU (2.5 equiv), and 4-ethylmorpholine (2.5 equiv) in DMF for 3.5 h at room temperature to yield the HMPA-ChemMatrix rein. The first amino acid Fmoc-Asp(OtBu) (5 equiv) was coupled with MSNT (5 equiv) and 1-methylimidazole (3.75 equiv) in DCM for 1 h. The Fmoc group was deprotected by treatment with 20% piperidine in DMF for 15 min. Each amino acid (4 equiv) was coupled using DIC (4 equiv) and HOBt (4 equiv) in DMF for 1 h. For the coupling of **L-9** and **D-9**, 1.3 equiv of amino acid, DIC, and HOBt were used. The peptides were cleaved off from the resin by treatment with TFA/H_2_O/TIPS (95/2.5/2.5) for 2 h. The resin was filtered off and the filtrate was concentrated *in vacuo* to yield crude pentapeptides containing a δ-selenolysine residue. The crude peptides (3 μmol) were treated with 0.2 M MeONH_2_·HCl, 6 M Gn·HCl, 0.2 M phosphate buffer at pH 4.5 (550 μL) for 17 h. Diselenides **L-11**/**D-11** were purified by RP-HPLC [CAPCELLPAK C18 (10 mm × 250 mm), solvent A:solvent B = 95:5 to 75:25 over 30 min at a flow rate of 2 mL/min) and lyophilized. Purified diselenides were dissolved in 25 mM DTT, 6 M Gn·HCl, 0.2 M phosphate buffer at pH 6.5 (582 μL), purged with Ar for 3 min, and stood for 10 min. Then, a solution of 200 mM TCEP, 6 M Gn·HCl, 0.2 M phosphate buffer at pH 5.4 (632 μL) was added to the solution and stood overnight. RP-HPLC purification [CAPCELLPAK C18 (10 mm × 250 mm), solvent A:solvent B = 95:5 to 70:30 over 45 min at a flow rate of 2 mL/min) and lyophilization yielded the pentapeptide **L-12**/**D-12**, which were analyzed by RP-HPLC [CAPCELLPAK C18 (4.6 mm × 150 mm), solvent A:solvent B = 98:2 to 80:20 over 15 min at a flow rate of 1 mL/min] by co-injection with pentapeptide samples synthesized separately using Fmoc-L-Lys(Boc) or Fmoc-D-Lys(Boc).

### 3.8 Preparation of hydrazine resin

2-Chlorotrityl chloride resin (76.9 mg, 100 μmol, 1.30 mmol/g loading) was washed with DMF × 2, DCM × 5, and DMF × 3, and was swollen in DCM (1.385 mL) for 15 min at 0°C. A solution of 9-fluorenylmethyl carbazate (25.7 mg, 100 μmol) in DMF (1.77 mL) and DCM (0.385 mL) was added to the suspension, followed by DIEA (44.3 μL, 250 μmol, 2.5 equiv). The suspension was first shaken for 5 min at 0°C and then for an additional 2.5 h at room temperature. MeOH (81 μL, 2.0 mmol, 20 equiv) and DIEA (340 μL, 2.0 mmol, 20 equiv) were added to the suspension and shaken for 1 h. The resin was filtered off and washed with DCM/MeOH/DIEA = 17/2/1 (2 mL × 3), DCM × 5, DMF × 5, water × 5, DMF × 5, DCM × 5, DMF × 5, MeOH × 3, and Et_2_O × 3 and dried in a vacuum desiccator overnight. The dried resin was swollen in DMF/DCM = 1/1 for 10 min, washed with DCM × 5, and DMF × 5, and treated with 20% piperidine in DMF (4 mL) for 15 min. Fmoc quantification suggested that the amount of loaded hydrazine was 42 μmol (0.52 mmol/g loading).

### 3.9 Fmoc SPPS of Ub (49–76)-hydrazide resin

A solution of Fmoc-Gly (420 μmol, 10 equiv), DIC (65 μL, 10 equiv), and OxymaPure (59.7 mg, 10 equiv) in NMP (1,050 μL) was preactivated for 3 min and added to hydrazine-2-chlorotrityl resin (42 μmol). The suspension was shaken for 20 min at 60°C. The coupling of Fmoc-Gly was performed twice. After washing the resin with DMF × 5, it was treated with Ac_2_O (0.1 mL), HOBt (4.7 mg), and DIEA (47 μL) in NMP (2.1 mL) for 10 min at room temperature. The resin was then treated with 20% piperidine in DMF for 15 min at room temperature, and the amount of Gly loading was estimated to be 26 μmol. Peptide elongation was manually carried out according to the general steps below. Coupling step: Fmoc-AA (420 μmol, 10 equiv), DIC (65 μL, 10 equiv), OxymaPure (59.7 mg, 10 equiv) in NMP (1,050 μL) was preactivated for 3min. The solution was added to the resin and shaken for 20 min at 60°C. The coupling of Fmoc-His(Trt) was performed for 60 min at room temperature. Dipeptides Fmoc-Leu-Ser(psiMe, Mepro) and Fmoc-Asp(OtBu)-(Dmb)Gly were used for Leu56—Ser57 and Asp52—Gly53, respectively. Dipeptides were coupled via double coupling using 4 equiv followed by using 2 equiv. Deprotection step: 20% piperidine in DMF was added to the resin and sonicated with ultrasound for 30 s and 1 min.

### 3.10 Ub (46C^Acm^—76)-α-hydrazide (13)

Approximately 1/4th of the aforementioned Ub (49–76)-hydrazine resin was treated with 20% piperidine in DMF (0.5 mL) for 15 min at room temperature, and the amount of the peptide was estimated to be 3.9 μmol. Peptide elongation was manually carried out according to the general steps given below. Coupling step: Fmoc-Lys (Boc) and Fmoc-Gly (50 μmol), DIC (7.75 μL, 10 equiv), and OxymaPure (7.1 mg, 10 equiv) in NMP (250 μL) were preactivated for 3min, and the solution was added to the resin and shaken for 20 min at 60°C; Deprotection step: 20% piperidine in DMF was added to the resin and sonicated with ultrasound for 30 s and 1 min. Finally, Boc-Cys (Acm) (50 μmol), DIC (7.75 μL, 10 equiv), and OxymaPure (7.1 mg, 10 equiv) in NMP (250 μL) were preactivated for 3min, and the solution was added to the resin and shaken for 60 min at room temperature. The resin was washed with DMF × 5, DCM × 5, DMF × 5, MeOH × 3, and Et_2_O × 3 and dried in a vacuum desiccator overnight. The dried resin was treated with a mixture comprising TFA (950 μL), TIPS (25 μL), water (25 μL) and shaken for 2 h at room temperature. The resin was filtered off, and the filtrate was added dropwise to ice-cold Et_2_O (10 mL). The precipitate was collected via centrifugation. The remaining resin was washed with TFA (1 mL), and the washing was treated in the same way. The precipitate was washed twice with ice-cold Et_2_O and dried in a vacuum desiccator overnight. The dried crude peptide was purified by RP-HPLC [Proteonavi (10 mm × 250 mm), solvent A:solvent B = 85:15 to 65:35 over 100 min at a flow rate of 2 mL/min] and lyophilized to yield Ub (46C^Acm^–76)-α-hydrazide **13** (6.5 mg, 1.8 μmol, 17% calculated using 1/4th of the starting hydrazine-2-chlorotrityl resin) as a white fluffy solid.; Analytical HPLC conditions: Proteonavi (4.6 mm × 150 mm), solvent A:solvent B = 85:15 to 55:45 over 15 min at a flow rate of 1 mL/min.; ESI-MS: calcd. for C_152_H_258_N_50_O_48_S 3586.1, obsd. 3586.1 ± 0.2. *m/z* calcd. [M+3H]^3+^ 1196.4, [M+4H]^4+^ 897.5, [M+5H]^5+^ 718.2, [M+6H]^6+^ 598.7, [M+7H]^7+^ 513.3; obsd. [M+3H]^3+^ 1196.3, [M+4H]^4+^ 897.6, [M+5H]^5+^ 718.2, [M+6H]^6+^ 598.7, [M+7H]^7+^ 513.3.

### 3.11 Ub (46C^Acm^—76)-α-MPAA (14)

Ub (46C^Acm^–76)-α-hydrazide **13** (5.1 mg, 1.4 μmol) was dissolved in 0.2 M MPAA, 6 M Gn·HCl, 0.2 M phosphate buffer at pH 3.0 (286 μL), and 0.1 M aqueous acetylacetone (143 μL, 14.2 μmol, 10 equiv) was added. The suspension was incubated overnight at room temperature. It was then diluted with 1 M Gn·HCl, 33 mM phosphate buffer at pH 3.0 (4 mL), filtered through Millex LCR syringe filter, and purified by RP-HPLC [Proteonavi (10 mm × 250 mm), solvent A:solvent B = 80:20 to 50:50 over 60 min at a flow rate of 2 mL/min]. Fractions containing the desired product were collected and lyophilized to yield Ub (46C^Acm^–76)-α-MPAA **14** (3.7 mg, 0.99 μmol, 70%) as a white fluffy solid.; Analytical HPLC conditions: Proteonavi (4.6 mm × 150 mm), solvent A:solvent B = 80:20 to 50:50 over 15 min at a flow rate of 1 mL/min.; ESI-MS: calcd. for C_160_H_262_N_48_O_50_S_2_ 3722.2, obsd. 3722.4 ± 0.2. *m/z* calcd. [M+3H]^3+^ 1241.7, [M+4H]^4+^ 931.6, [M+5H]^5+^ 745.5, [M+6H]^6+^ 621.4, [M+7H]^7+^ 532.8; obsd. [M+3H]^3+^ 1241.7, [M+4H]^4+^ 931.6, [M+5H]^5+^ 745.5, [M+6H]^6+^ 621.4, [M+7H]^7+^ 532.8.

### 3.12 Ub (46C^Acm^–48K^Sez^–76)-α-hydrazide (15)

Approximately 1/4th of the aforementioned Ub (49–76)-hydrazine resin was treated with 20% piperidine in DMF (0.5 mL) for 15 min at room temperature, and the amount of the peptide was estimated to be 4.4 μmol. Fmoc-Lys(Sez) **L-9** (5.6 mg, 10 μmol, 2 equiv), DIC (1.55 μL, 2 equiv), and OxymaPure (1.4 mg, 2 equiv) in NMP (100 μL) were preactivated for 3min, and the solution was added to the resin and shaken for 20 min at 60°C. The coupling was performed twice using the same conditions. Peptide elongation using Fmoc-Gly (double coupling) and Boc-Cys(Acm) was carried out as mentioned above for the synthesis of **13**. The resin was washed with DMF × 5, DCM × 5, DMF × 5, MeOH × 3, and Et_2_O × 3 and dried in a vacuum desiccator overnight. The dried resin was treated with a mixture comprising TFA (950 μL), TIPS (25 μL), water (25 μL), MeONH_2_·HCl (2.1 mg, 5 equiv) and allowed to shake for 2 h at room temperature. The crude peptide was obtained by ice-cold Et_2_O treatment, using the same method as that for **13**. The dried crude peptide was purified by RP-HPLC [Proteonavi (10 mm × 250 mm), solvent A:solvent B = 85:15 to 65:35 over 100 min at a flow rate of 2 mL/min] and lyophilized to yield Ub (46C^Acm^–48K^Sez^–76)-α-hydrazide **15** (3.7 mg, 1.0 μmol, 10% calculated based on the 1/4th of the starting hydrazine-2CT resin) as a white fluffy solid.; Analytical HPLC conditions: Proteonavi (4.6 mm × 150 mm), solvent A:solvent B = 85:15 to 55:45 over 15 min at a flow rate of 1 mL/min.; ESI-MS: calcd. for C_153_H_258_N_50_O_48_SSe 3677.0, obsd. 3677.3 ± 0.4. *m/z* calcd. [M+3H]^3+^ 1266.7, [M+4H]^4+^ 920.3, [M+5H]^5+^ 736.4, [M+6H]^6+^ 613.8, [M+7H]^7+^ 526.3; obsd. [M+3H]^3+^ 1226.5, [M+4H]^4+^ 920.4, [M+5H]^5+^ 736.5, [M+6H]^6+^ 613.9, [M+7H]^7+^ 526.4.

### 3.13 One-pot selenazolidine deprotection–δ-selenolysine-mediated ligation–deselenization

Ub (46C^Acm^–48K^Sez^–76)-α-hydrazide **15** (0.6 mg, 0.16 μmol) was dissolved in a degassed solution of 3 mM CuCl_2_ (1.5 equiv), 10 mM MeONH_2_·HCl (5 equiv) in 6 M Gn·HCl, 0.2 M phosphate (81 μL, pH 6). The resulting suspension was vortexed for 30 min. The LC/MS analysis results showed the formation of diselenide **16**. The solution was degassed for 5 min with Ar bubbling, and a solution of Ub (46C^Acm^–76)-α-MPAA **14** (0.8 mg, 0.22 μmol) in 10 mM TCEP (5 equiv), 6 M Gn·HCl, and 0.2 M phosphate (81 μL, pH 6) was added. The resulting solution was allowed to stand for 2 h. The LC/MS analysis results showed the formation of isopeptide **18**. The solution was degassed for more than 10 min with Ar bubbling. TCEP (2.4 mg, 8.2 μmol, 50 equiv) and DTT (1.2 mg, 8.2 μmol, 50 equiv) were added to the degassed solution and the pH was adjusted to 5 with 1 M NaOH. The resulting solution was again bubbled with Ar for 5 min and allowed to stand overnight. The solution was diluted with aqueous 0.04% TFA (0.8 mL) and purified by RP-HPLC [Proteonavi (10 mm × 150 mm), solvent A:solvent B = 95:5 to 87:13 over 5 min then to 63:37 over 100 min at a flow rate of 2 mL/min] and lyophilized to yield Ub [46C^Acm^–48K{Ub (46C^Acm^–76)}–76]-α-hydrazide **21** (0.6 mg, 53%) as a white fluffy solid.; Analytical HPLC conditions: Proteonavi (4.6 mm × 150 mm), solvent A:solvent B = 80:20 to 60:40 over 15 min at a flow rate of 1 mL/min.

Compound **16**: calcd. for C_304_H_514_N_100_O_96_S_2_Se_2_ 7328.0, obsd. 7328.5 ± 0.5. *m/z* calcd. [M+6H]^6+^ 1222.3, [M+7H]^7+^ 1047.9, [M+8H]^8+^ 917.0, [M+9H]^9+^ 815.2, [M+10H]^10+^ 733.8, [M+11H]^11+^ 667.2, [M+12H]^12+^ 611.7, [M+13H]^13+^ 564.7, [M+14H]^14+^ 524.4; obsd. [M+6H]^6+^ 1222.4, [M+7H]^7+^ 1048.0, [M+8H]^8+^ 917.1, [M+9H]^9+^ 815.3, [M+10H]^10+^ 733.9, [M+11H]^11+^ 667.3, [M+12H]^12+^ 611.7, [M+13H]^13+^ 564.7, [M+14H]^14+^ 524.4.

Compound **18**: calcd. for C_304_H_512_N_98_O_96_S_2_Se 7219.0, obsd. 7220.0 ± 0.9. *m/z* calcd. [M+5H]^5+^ 1444.8, [M+6H]^6+^ 1204.1, [M+7H]^7+^ 1032.2, [M+8H]^8+^ 903.3, [M+9H]^9+^ 803.1, [M+10H]^10+^ 722.9, [M+11H]^11+^ 657.3, [M+12H]^12+^ 602.6; obsd. [M+5H]^5+^ 1444.9, [M+6H]^6+^ 1204.3, [M+7H]^7+^ 1032.2, [M+8H]^8+^ 903.6, [M+9H]^9+^ 803.4, [M+10H]^10+^ 723.1, [M+11H]^11+^ 657.3, [M+12H]^12+^ 602.6.

Compound **21**: calcd. for C_304_H_512_N_98_O_96_S_2_ 7140.1, obsd. 7140.0 ± 0.5. *m/z* calcd. [M+5H]^5+^ 1429.0, [M+6H]^6+^ 1191.0, [M+7H]^7+^ 1021.0, [M+8H]^8+^ 893.5, [M+9H]^9+^ 794.3, [M+10H]^10+^ 715.0, [M+11H]^11+^ 650.1, [M+12H]^12+^ 596.0, [M+13H]^13+^ 550.2; obsd. [M+5H]^5+^ 1428.9, [M+6H]^6+^ 1191.0, [M+7H]^7+^ 1021.0, [M+8H]^8+^ 893.5, [M+9H]^9+^ 794.4, [M+10H]^10+^ 715.0, [M+11H]^11+^ 650.2, [M+12H]^12+^ 596.0, [M+13H]^13+^ 550.2.

### 3.14 Trypsin digestion of isopeptide (21)

An aliquot of isopeptide **21** was dissolved in 50 mM ammonium bicarbonate buffer (pH 8.0, 20 μL), and a trypsin solution (20 μg/200 μL, 0.3 μL) was added. The resulting solution was incubated overnight at 37°C and analyzed by LC/MS [CAPCELLPAK C18 (Osaka soda, 5 μm, 2 × 150 mm), solution A:solution B = 98:2 to 40:60 over 10 min at a flow rate of 0.2 mL/min]. ESI-MS: (Peak a in Supplementary Figure S8) calcd. for C_28_H_48_N_10_O_12_ 716.3, obsd. 716.3. *m/z* calcd. [M + H]^+^ 717.4, [M+2H]^2+^ 359.2; obsd. [M + H]^+^ 717.5, [M+2H]^2+^ 359.2; (Peak b in Supplementary Figure S8) calcd. for C_46_H_79_N_17_O_18_S 1189.6, obsd. 1189.8 *m/z* calcd. [M+3H]^3+^ 397.5; obsd. [M+3H]^3+^ 397.6; (Peak c in Supplementary Figure S8) calcd. for C_47_H_76_N_12_O_17_ 1080.5, obsd. 1080.6. *m/z* calcd. [M + H]^+^ 1081.6, [M+2H]^2+^ 541.3; obsd. [M + H]^+^ 1081.6, [M+2H]^2+^ 541.3; (Peak d in Supplementary Figure S8) calcd. for C_47_H_82_N_14_O_14_ 1066.6, obsd. 1066.6. *m/z* calcd. [M + H]^+^ 1067.6, [M+2H]^2+^ 534.3, [M+3H]^3+^ 356.5; obsd. [M + H]^+^ 1067.7, [M+2H]^2+^ 534.3, [M+3H]^3+^ 356.6.

## 4 Conclusion

We have developed a δ-selenolysine-mediated pathway for the formation of isopeptide bonds. *N*-α-Fmoc-δ-selenolysine, with its side chain protected as selenazolidine, was synthesized in both L- and D-forms starting from commercially available racemic DL-δ-hydroxy-DL-lysine. Substitution of δ-mesylate by KSeCN and enzymatic optical resolution using L- or D-aminoacylase successfully provided the desired **L-9** and **D-9**, respectively. We prepared both L- and D-forms of the δ-selenolysine derivative because the L-form is essential for the synthesis of native ubiquitinated protein probes, while the D-form is valuable for the synthesis of the mirror-image protein that is required for racemic protein crystallography. Then, we examined the isopeptide bond formation mediated by δ-selenolysine between ubiquitin (46–76)-α-thioester and ubiquitin (46–76)-α-hydrazide containing a δ-selenolysine residue at position 48. After thoroughly analyzing the byproducts, we established a one-pot protocol involving selenazolidine deprotection, δ-selenolysine-mediated isopeptide chemical ligation, and deselenization. We anticipate that the δ-selenolysine-mediated isopeptide bond formation reported here may prove helpful for the synthesis of ubiquitinated proteins with native cysteine residues, such as glycoproteins, as deselenization proceeds without desulfurization of cysteine residues. We are currently studying the synthesis of di-ubiquitins using δ-selenolysine-mediated isopeptide bond formation in our laboratory and will report the results in due course.

## Data Availability

The original contributions presented in the study are included in the article/Supplementary Material, further inquiries can be directed to the corresponding author.

## References

[B1] Bacsa B. Horváti K. Bõsze S. Andreae F. Kappe C. O. (2008). Solid-phase synthesis of difficult peptide sequences at elevated temperatures: a critical comparison of microwave and conventional heating technologies. J. Org. Chem. 73 (19), 7532–7542. 10.1021/jo8013897 18729524

[B2] Bavikar S. N. Spasser L. Haj-Yahya M. Karthikeyan S. V. Moyal T. Kumar K. S. A. (2012). Chemical synthesis of ubiquitinated peptides with varying lengths and types of ubiquitin chains to explore the activity of deubiquitinases. Angew. Chem. Int. Ed. 51 (3), 758–763. 10.1002/anie.201106430 22131237

[B3] Chatterjee C. Mcginty R. K. Pellois J. P. Muir T. W. (2007). Auxiliary-mediated site-specific peptide ubiquitylation. Angew. Chem. Int. Ed. 46 (16), 2814–2818. 10.1002/anie.200605155 17366504

[B4] Chaugule V. K. Walden H. (2016). Specificity and disease in the ubiquitin system. Biochem. Soc. Trans. 44 (1), 212–227. 10.1042/BST20150209 26862208 PMC5264512

[B5] Dardashti R. N. Kumar S. Sternisha S. M. Reddy P. S. Miller B. G. Metanis N. (2020). Selenolysine: a new tool for traceless isopeptide bond formation. Chem. Eur. J. 26 (22), 4952–4957. 10.1002/chem.202000310 31960982 PMC7184786

[B6] El Oualid F. Merkx R. Ekkebus R. Hameed D. S. Smit J. J. De Jong A. (2010). Chemical synthesis of ubiquitin, ubiquitin-based probes, and diubiquitin. Angew. Chem. Int. Ed. 49 (52), 10149–10153. 10.1002/anie.201005995 PMC3021723 21117055

[B7] Erlich L. A. Kumar K. S. A. Haj-Yahya M. Dawson P. E. Brik A. (2010). N-methylcysteine-mediated total chemical synthesis of ubiquitin thioester. Org. Biomol. Chem. 8 (10), 2392–2396. 10.1039/c000332h 20448897 PMC3183995

[B8] Faggiano S. Alfano C. Pastore A. (2016). The missing links to link ubiquitin: methods for the enzymatic production of polyubiquitin chains. Anal. Biochem. 492, 82–90. 10.1016/j.ab.2015.09.013 26470940

[B9] Flood D. T. Hintzen J. C. J. Bird M. J. Cistrone P. A. Chen J. S. Dawson P. E. (2018). Leveraging the Knorr pyrazole synthesis for the facile generation of thioester surrogates for use in native chemical ligation. Angew. Chem. Int. Ed. 57 (36), 11634–11639. 10.1002/anie.201805191 PMC6126375 29908104

[B10] Gui W. Davidson G. A. Zhuang Z. (2021). Chemical methods for protein site-specific ubiquitination. RSC Chem. Biol. 2 (2), 450–467. 10.1039/d0cb00215a 34381999 PMC8323803

[B11] Haj-Yahya M. Ajish Kumar K. S. Erlich L. A. Brik A. (2010). Protecting group variations of δ-mercaptolysine useful in chemical ubiquitylation. Biopolymers 94 (4), 504–510. 10.1002/bip.21384 20564006

[B12] Haj-Yahya M. Fauvet B. Herman-Bachinsky Y. Hejjaoui M. Bavikar S. N. Karthikeyan S. V. (2013). Synthetic polyubiquitinated α-Synuclein reveals important insights into the roles of the ubiquitin chain in regulating its pathophysiology. Proc. Nat. Acad. Sci. U. S. A. 110 (44), 17726–17731. 10.1073/Pnas.1315654110 PMC3816408 24043770

[B13] Hershko A. Ciechanover A. (1998). The ubiquitin system. Annu. Rev. Biochem. 67, 425–479. 10.1146/Annurev.Biochem.67.1.425 9759494

[B14] Huang Y. C. Chen C. C. Li S. J. Gao S. Shi J. Li Y. M. (2014). Facile synthesis of C-terminal peptide hydrazide and thioester of NY-ESO-1 (A39-A68) from an Fmoc-hydrazine 2-chlorotrityl chloride resin. Tetrahedron 70 (18), 2951–2955. 10.1016/j.tet.2014.03.022

[B15] Huppelschoten Y. van der Heden van Noort G. J. (2022). State of the art in (semi-)synthesis of ubiquitin- and ubiquitin-like tools. Semin. Cell Dev. Biol. 132, 74–85. 10.1016/j.semcdb.2021.11.025 34961664

[B16] Izumi M. Araki H. Tominaga M. Okamoto R. Kajihara Y. (2020). Chemical synthesis of ubiquitinated high-mannose-type N-glycoprotein CCL1 in different folding states. J. Org. Chem. 85 (24), 16024–16034. 10.1021/acs.joc.0c01766 32985191

[B17] Komander D. Rape M. (2012). The ubiquitin code. Annu. Rev. Biochem. 81 (1), 203–229. 10.1146/annurev-biochem-060310-170328 22524316

[B18] Kumar K. S. A. Haj-Yahya M. Olschewski D. Lashuel H. A. Brik A. (2009). Highly efficient and chemoselective peptide ubiquitylation. Angew. Chem. Int. Ed. 48 (43), 8090–8094. 10.1002/anie.200902936 19780082

[B19] Kumar K. S. A. Spasser L. Erlich L. A. Bavikar S. N. Brik A. (2010). Total chemical synthesis of di-ubiquitin chains. Angew. Chem. Int. Ed. 49 (48), 9126–9131. 10.1002/anie.201003763 20815002

[B20] Lander A. J. Jin Y. Luk L. Y. P. (2023). D-peptide and D-protein technology: recent advances, challenges, and opportunities. ChemBioChem 24 (4), e202200537. 10.1002/cbic.202200537 36278392 PMC10805118

[B21] Mali S. M. Singh S. K. Eid E. Brik A. (2017). Ubiquitin signaling: chemistry comes to the rescue. J. Am. Chem. Soc. 139 (14), 4971–4986. 10.1021/jacs.7b00089 28328208

[B22] Malins L. R. Payne R. J. (2012). Synthesis and utility of β-selenol-phenylalanine for native chemical ligation-deselenization chemistry. Org. Lett. 14 (12), 3142–3145. 10.1021/ol3012265 22642500

[B23] Merkx R. De Bruin G. Kruithof A. van den Bergh T. Snip E. Lutz M. (2013). Scalable synthesis of γ-thiolysine starting from lysine and a side by side comparison with δ-thiolysine in non-enzymatic ubiquitination. Chem. Sci. 4 (12), 4494–4498. 10.1039/c3sc51599k

[B24] Metanis N. Keinan E. Dawson P. E. (2010). Traceless ligation of cysteine peptides using selective deselenization. Angew. Chem. Int. Ed. 49 (39), 7049–7053. 10.1002/anie.201001900 PMC4459706 20715234

[B25] Morishita Y. Kaino T. Okamoto R. Izumi M. Kajihara Y. (2015). Synthesis of D,L-amino acid derivatives bearing a thiol at the β-position and their enzymatic optical resolution. Tetrahedron Lett. 56 (47), 6565–6568. 10.1016/j.tetlet.2015.10.015

[B26] Mulder M. P. C. Witting K. F. Ovaa H. (2020). Cracking the ubiquitin code: the ubiquitin toolbox. Curr. Issues Mol. Biol. 37, 1–20. 10.21775/cimb.037.001 31674341

[B27] Pan M. Gao S. Zheng Y. Tan X. Lan H. Tan X. (2016). Quasi-racemic X-ray structures of K27-linked ubiquitin chains prepared by total chemical synthesis. J. Am. Chem. Soc. 138 (23), 7429–7435. 10.1021/jacs.6b04031 27268299

[B28] Reddy P. S. Dery S. Metanis N. (2016). Chemical synthesis of proteins with non-strategically placed cysteines using selenazolidine and selective deselenization. Angew. Chem. Int. Ed. 55 (3), 992–995. 10.1002/anie.201509378 26636774

[B29] Seenaiah M. Jbara M. Mali S. M. Brik A. (2015). Convergent versus sequential protein synthesis: the case of ubiquitinated and glycosylated H2B. Angew. Chem. Int. Ed. 54 (42), 12374–12378. 10.1002/anie.201503309 26079184

[B30] Swatek K. N. Komander D. (2016). Ubiquitin modifications. Cell Res. 26 (4), 399–422. 10.1038/cr.2016.39 27012465 PMC4822133

[B31] Tang S. Liang L. J. Si Y. Y. Gao S. Wang J. X. Liang J. (2017). Practical chemical synthesis of atypical ubiquitin chains by using an isopeptide-linked Ub isomer. Angew. Chem. Int. Ed. 56 (43), 13333–13337. 10.1002/anie.201708067 28873270

[B32] Virdee S. Kapadnis P. B. Elliott T. Lang K. Madrzak J. Nguyen D. P. (2011). Traceless and site-specific ubiquitination of recombinant proteins. J. Am. Chem. Soc. 133 (28), 10708–10711. 10.1021/ja202799r 21710965 PMC3135006

[B33] Wang J. Gao S. Pan M. Zheng Y. Huang Y. Zheng Q. (2016). Monomer/oligomer quasi-racemic protein crystallography. J. Am. Chem. Soc. 138 (43), 14497–14502. 10.1021/jacs.6b09545 27768314

[B34] Wang X. Corcilius L. Premdjee B. Payne R. J. (2020). Synthesis and utility of β-selenophenylalanine and β-selenoleucine in diselenide-selenoester ligation. J. Org. Chem. 85 (3), 1567–1578. 10.1021/acs.joc.9b02665 31840993

[B35] Yan B. J. Ye L. Z. Xu W. L. Liu L. (2017). Recent advances in racemic protein crystallography. Biorg. Med. Chem. 25 (18), 4953–4965. 10.1016/j.bmc.2017.05.020 28705433

[B36] Yang R. L. Pasunooti K. K. Li F. P. Liu X. W. Liu C. F. (2009). Dual native chemical ligation at lysine. J. Am. Chem. Soc. 131 (38), 13592–13593. 10.1021/ja905491p 19728708

[B37] Yeates T. O. Kent S. B. H. (2012). Racemic protein crystallography. Annu. Rev. Biophys. 41 (1), 41–61. 10.1146/annurev-biophys-050511-102333 22443988

[B38] Zhao Z. Metanis N. (2019). Copper-mediated selenazolidine deprotection enables one-pot chemical synthesis of challenging proteins. Angew. Chem. Int. Ed. 58 (41), 14610–14614. 10.1002/anie.201909484 31408267

