# Supplementary material for: Isopeptide bond formation mediated by δ-selenolysine for chemical ubiquitination
Source: Front Chem. 2023 Nov 21;11:1307254. doi: 10.3389/fchem.2023.1307254 (PMC10702597; doi:10.3389/fchem.2023.1307254)

Supplementary Material

Isopeptide bond formation mediated by δ-selenolysine for chemical ubiquitination

Tatsunari Akiyama, Yusuke Tanaka, Ryo Okamoto, Yasuhiro Kajihara, Masayuki Izumi*

*** Correspondence:** Masayuki Izumi: izumi@kochi-u.ac.jp

Contents

General and abbreviations S2

LC/MS analysis, HPLC analysis and purification S2

Supplementary scheme 1 and figures 1~8 S3

HRMS, ^{1}H and ^{13}C NMR spectra of products S12

1 General and abbreviations

All standard chemicals were purchased from Fujifilm Wako Pure Chemical Co., Tokyo Chemical Ind., Kishida Chemical Co. Ltd., Nacalai Tesque Inc., Kanto Chemical Co. Inc. δ-DL-hydroxy-DL-lysine hydrochloride was purchased from Bachem AG. L-Aminoacylase (Acylase H "Amano") and D-aminoacylase (D-Aminoacylase "Amano") were gifts from Amano Enzyme Inc. Fmoc-Phe, Fmoc-Lys(Boc), Fmoc-Pro, Fmoc-Ser(tBu), Fmoc-Asp(OtBu), Boc-Cys(Acm), Fmoc-Gly, Fmoc-Gln, Fmoc-Leu, Fmoc-Glu(OtBu), Fmoc-Arg(Pbf), Fmoc-Thr(tBu), Fmoc-Tyr(tBu), Fmoc-Asn(Trt), Fmoc-Ile, Fmoc-His(Boc), Fmoc-Val were purchased from Peptide Institute. Fmoc-Leu-Ser(psiMe,Mepro), Fmoc-Asp(OtBu)-(Dmb)Gly, 1-(mesitylene-2-sulfonyl)-3-nitro-*1H*-1,2,4-triazole (MSNT) were purchased from Merck. Fmoc-D-Lys(Boc), aminomethyl ChemMatrix resin, tris(2-carboxyethyl)phosphine hydrochloride (TCEP), 4-mercaptophenylacetic acid (MPAA), were purchased from Sigma-Aldrich Co. LLC. Trypsin mass spectrometry grade, D,L-dithiothreitol (DTT) were purchased from Fujifilm Wako Pure Chemical Co. 1-Hydroxy-benzotriazole (HOBt), ethyl cyano(hydroxyamino)acetate (OxymaPure), N,N'-diisopropylcarbodiimide (DIC), N,N-diisopropylethylamine (DIEA), triisopropylsilane (TIPS), N,N-Dimethylformamide (DMF), dichloromethane (DCM), N-Methyl-2-pyrrolidinone (NMP), trifluoroacetic acid (TFA), 4-hydroxymethylphenoxyacetic acid (HMPA), 2-(*1H*-Benzotriazol-1-yl)-1,1,3,3-tetramethyluronium tetrafluoroborate (TBTU), 2-chlorotrityl chloride (2-CTC) resin (1.30 mmol/mg resin) were purchased from Watanabe Chemical Ind. Ltd. Methoxyamine hydrochloride ($MeONH_2 \cdot HCl$), 9-fluorenylmethyl carbazate was purchased from Tokyo Chemical Ind. Guanidine hydrochloride (Gn·HCl), $Na_2HPO_4 \cdot 12H_2O$, diethyl ether (Et_2O) were purchased from Kishida Chemical Co. Ltd. ^1H and ^{13}C NMR spectra were recorded on a 400 MHz spectrometer (Bruker AVANCE III) or on a 500 MHz spectrometer (JEOL JNM-LA-500). All ^1H and ^{13}C chemical shifts were reported in parts per million (ppm) relative to TMS in $CDCl_3$ or CD_3OD. TLC analysis was conducted on Silica gel 60 F_{254} (Merck) and visualized by heating with 5% ninhydrin in EtOH. Silica gel column chromatography was performed using Silica gel 60N (Kanto Chemical Co. spherical, neutral). C18 silica gel chromatography was performed using COSMOSIL $75C_{18}$-OPN (Nacalai Tesque Inc.).

2 LC/MS analysis, HPLC analysis, and purification

Liquid chromatography-mass spectrometry (LC/MS) analyses were performed on a Bruker Daltonics amaZon-SL ion trap mass spectrometer equipped with an Agilent 1260 HPLC system. Gradient elution of water/0.1% formic acid (solvent A) versus 90% CH_3CN/0.1% formic acid (solvent B) was used. The eluent was monitored at 216 nm and 254 nm. For LC/MS analysis, CAPCELL PAK C18 (Osaka soda, 2.0 × 150 mm) and Proteonavi (Osaka soda, 2.0 × 150 mm) columns were used at the flow rate of 0.2 mL/min. Reversed-phase high-performance liquid chromatography (RP-HPLC) analyses were performed on a Waters 1525 HPLC system equipped with a 2489 UV-VIS detector. Gradient elution of water/0.04% TFA (solvent A) versus 90% CH_3CN/0.04% TFA (solvent B) was used, and the eluent was monitored at 216 nm. CAPCELL PAK C18 (Osaka soda, 4.6 × 150 mm) and Proteonavi (Osaka soda, 4.6 × 150 mm) columns were used at the flow rate of 1.0 mL/min. For semi-preparative HPLC, CAPCELL PAK C18 (Osaka soda, 10 × 250 mm) or Proteonavi (Osaka soda, 10 × 250 mm) columns were used at the flow rate of 2.0 mL/min. High resolution fourier transform ion cyclotron resonance mass spectrometry (HR-FTMS) analyses were performed on a Bruker Daltonics solariX 9.4T Fourier transform ion cyclotron mass spectrometer equipped with electrospray ionization source. High-

resolution electrospray ionization time-of-flight mass spectrometry (HR-TOFMS) analyses were performed on a JEOL JMS-T100LP AccuTOF spectrometer.

3 Supplementary Scheme and Figures

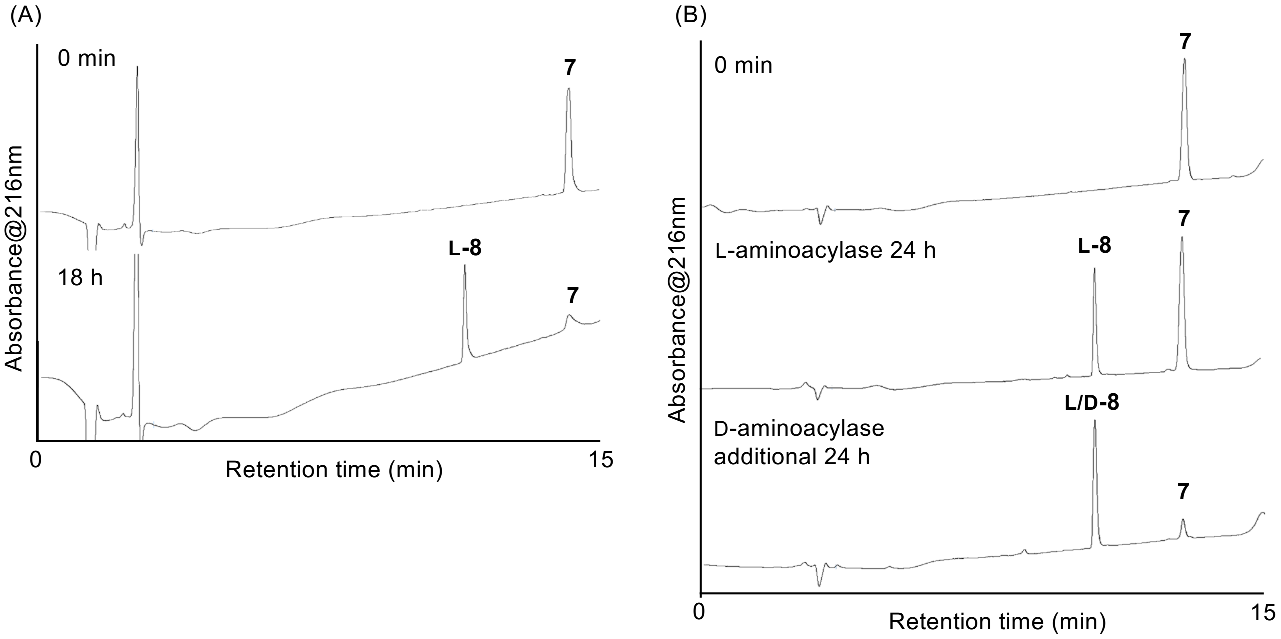

Supplementary Figure S1. HPLC chromatograms of enzymatic hydrolysis of **7**. **(A)** Isolated **7** after D-aminoacylase treatment was treated with L-aminoacylase. **(B)** Racemic **7** was treated with L-aminoacylase for 24 h then with D-aminoacylase in one pot for 24 h. Retention times of **7** and **8** in (A) and (B) are slightly different because the configuration of the HPLC pump is different.

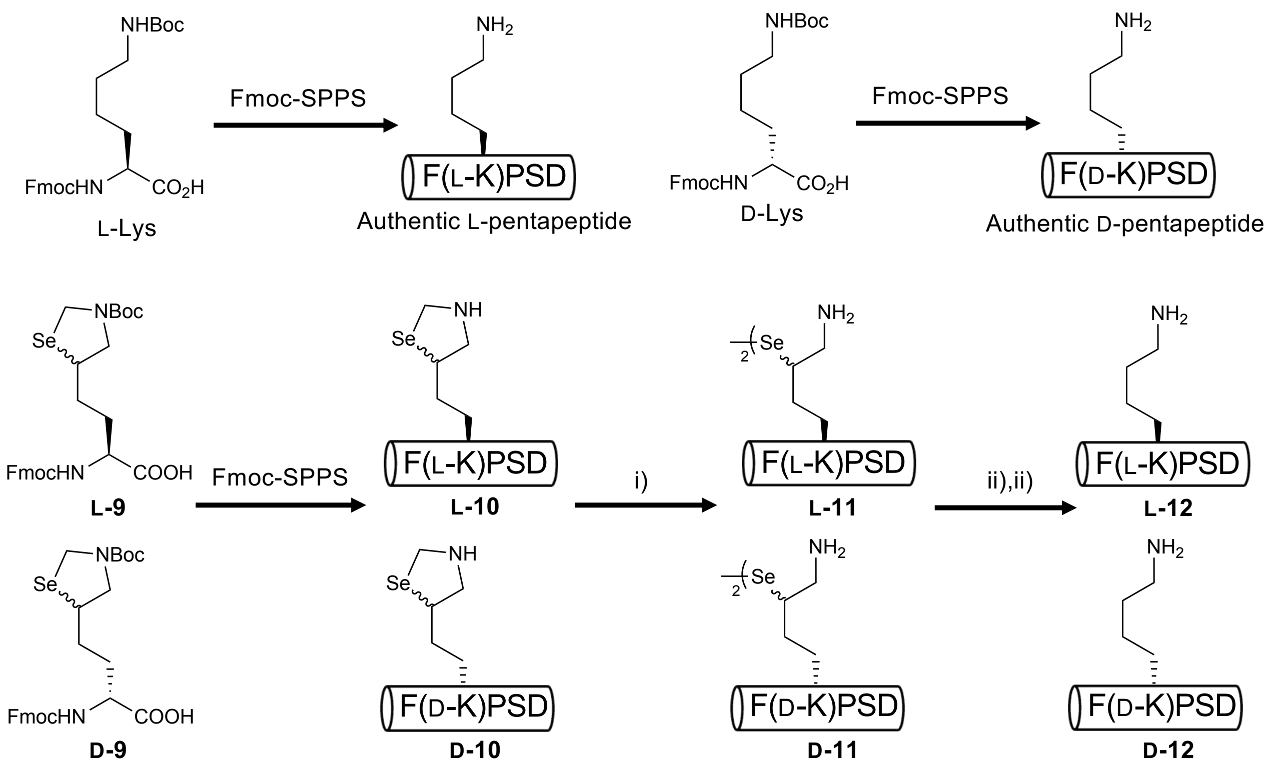

Supplementary Scheme S1. Synthesis of pentapeptide for the analysis of the absolute configuration of **L-9** and **D-9**. **i)** 0.2 M $MeONH_2 \cdot HCl$/6 M Gn·HCl/0.2 M Na_2HPO_4, pH 4.6; **ii)** 25 mM DTT/6 M Gn·HCl/0.2 M Na_2HPO_4, pH 6.5; **iii)** 0.2 M TCEP/6 M Gn·HCl/0.2 M Na_2HPO_4, pH 5.0.

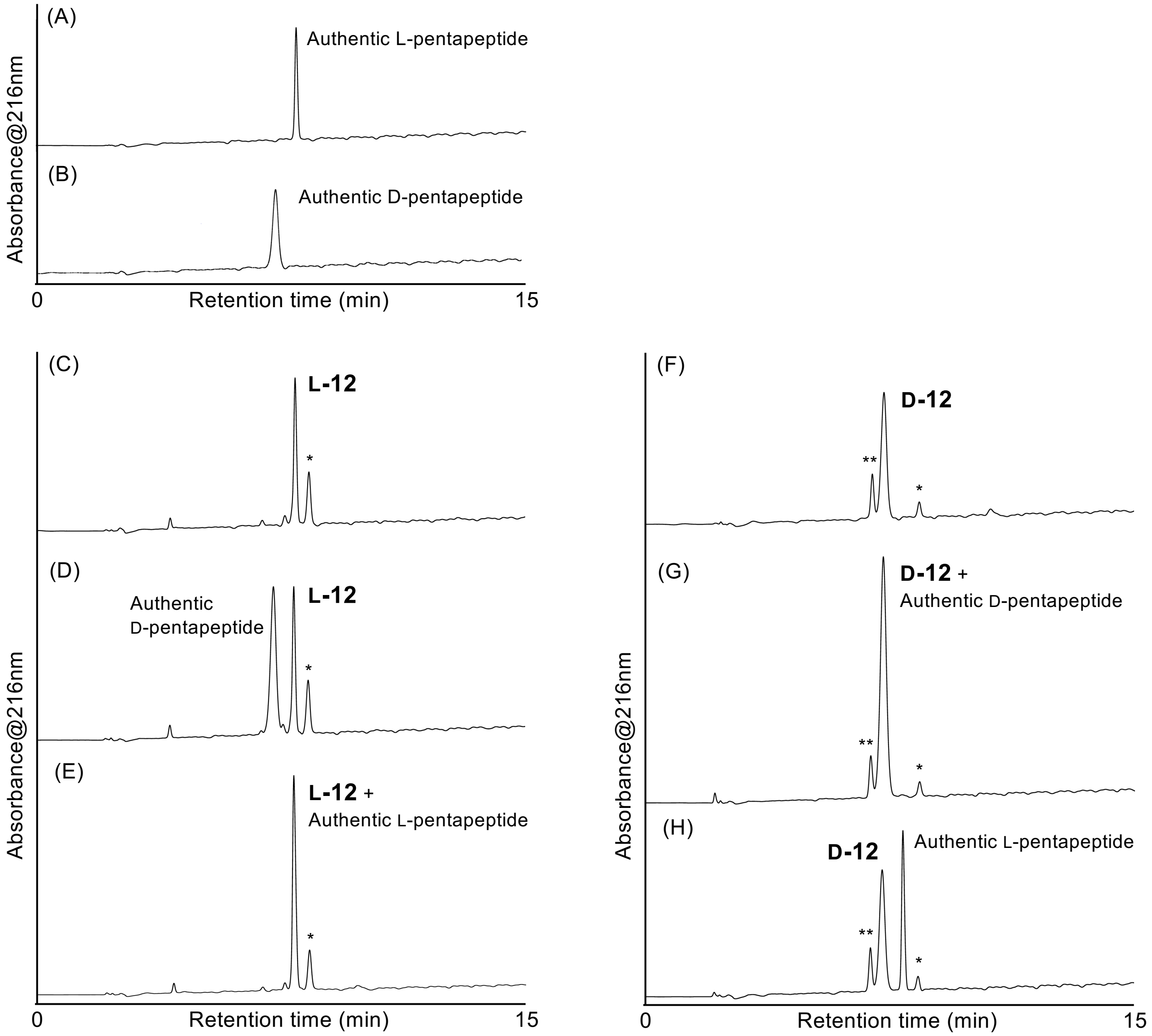

Supplementary Figure S2. HPLC chromatograms for the analysis of the absolute configuration of **L-9** and **D-9**. **(A)** Authentic pentapeptide F(L-K)PSD synthesized using commercial L-Lys(Boc). **(B)** Authentic pentapeptide F(D-K)PSD synthesized using commercial D-Lys(Boc). **(C)** Synthetic pentapeptide **L-12** using **L-9** after deselenization. **(D)** co-injection of **L-12** with authentic F(D-K)PSD . **(E)** co-injection of **L-12** with authentic F(L-K)PSD. **(F)** Synthetic pentapeptide **D-12** using **D-9** after deselenization. **(G)** co-injection of **D-12** with authentic F(D-K)PSD . **(H)** co-injection of **D-12** with authentic F(L-K)PSD. *Peak indicates TCEP=Se. **Peak indicates structurally unknown TCEP derivative.

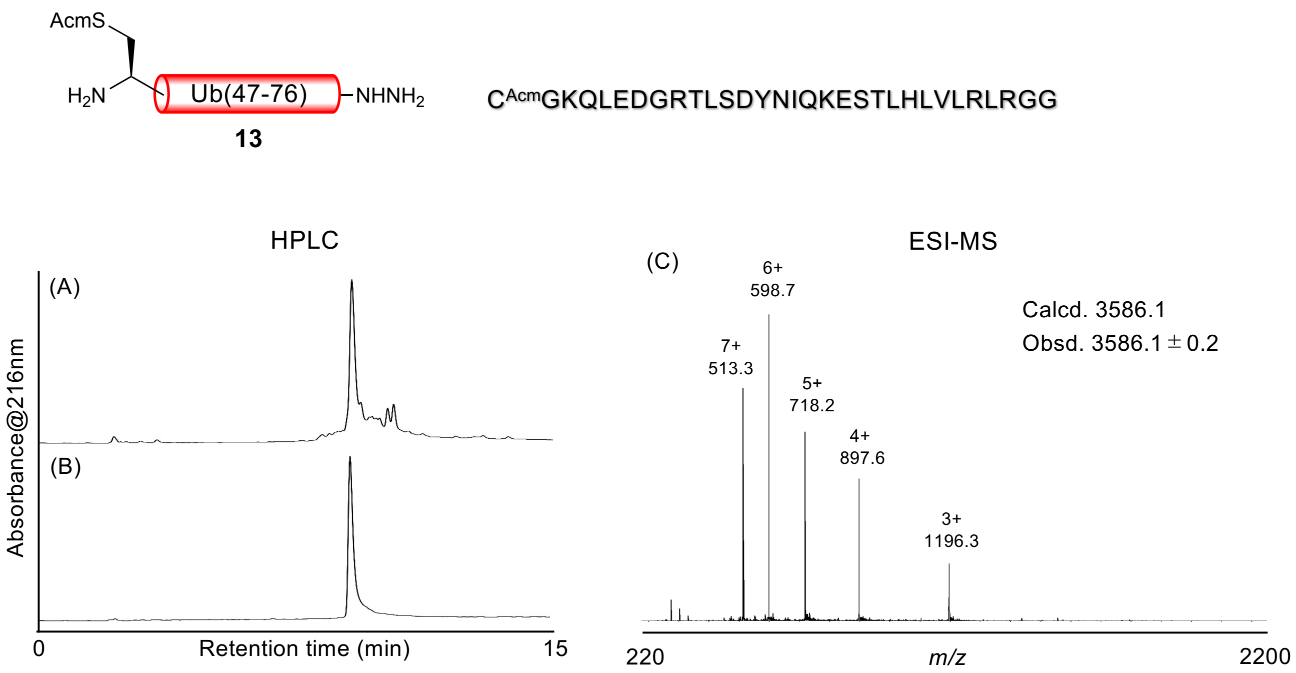

Supplementary Figure S3. Analytical HPLC chromatograms of Ub (46C^{Acm}–76)-α-hydrazide **13**. **(A)** crude peptide after global deprotection and cleavage. **(B)** purified **13**. **(C)** ESI-MS spectrum of purified **13**.

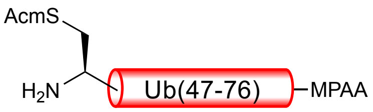

14

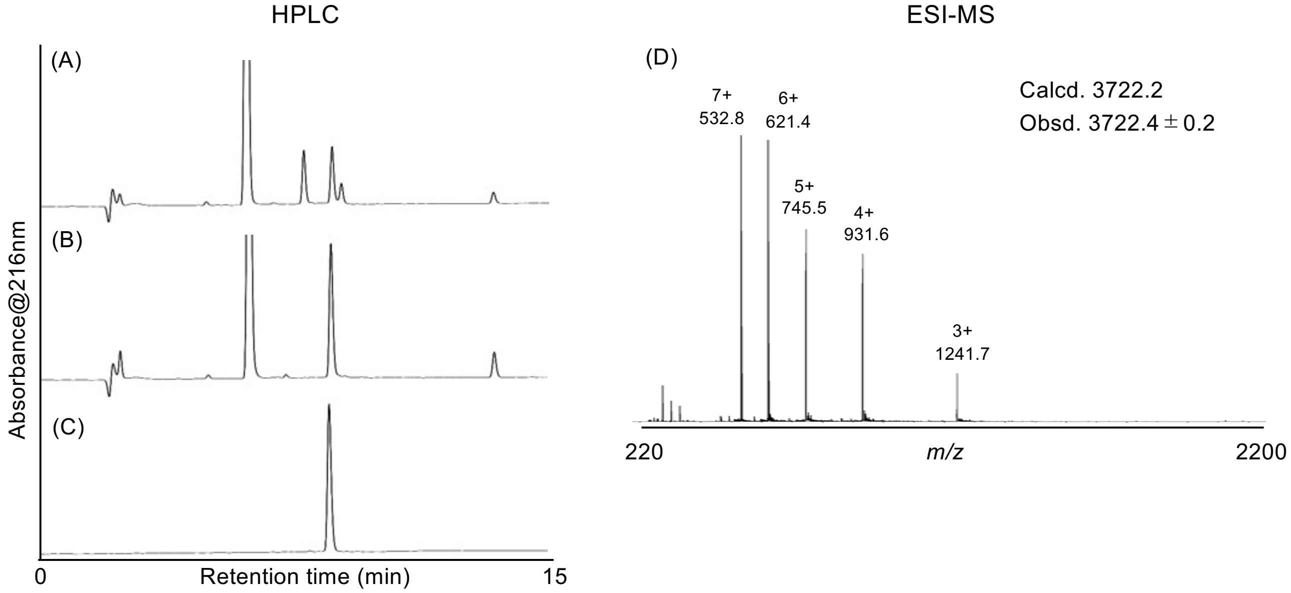

Supplementary Figure S4. Analytical HPLC chromatograms of conversion of Ub (46C^{Acm}–76)-α-hydrazide **13** to Ub (46C^{Acm}–76)-α-MPAA **14**. **(A)** reaction at 30 min. **(B)** reaction at 13 h. **(C)** purified **14**. **(D)** ESI-MS spectrum of purified **14**.

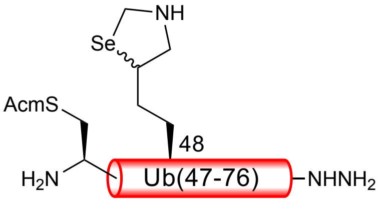

15

$C^{Acm}GK^{Sez}$QLEDGRTLSDYNIQKESTLHLVLRLRGG

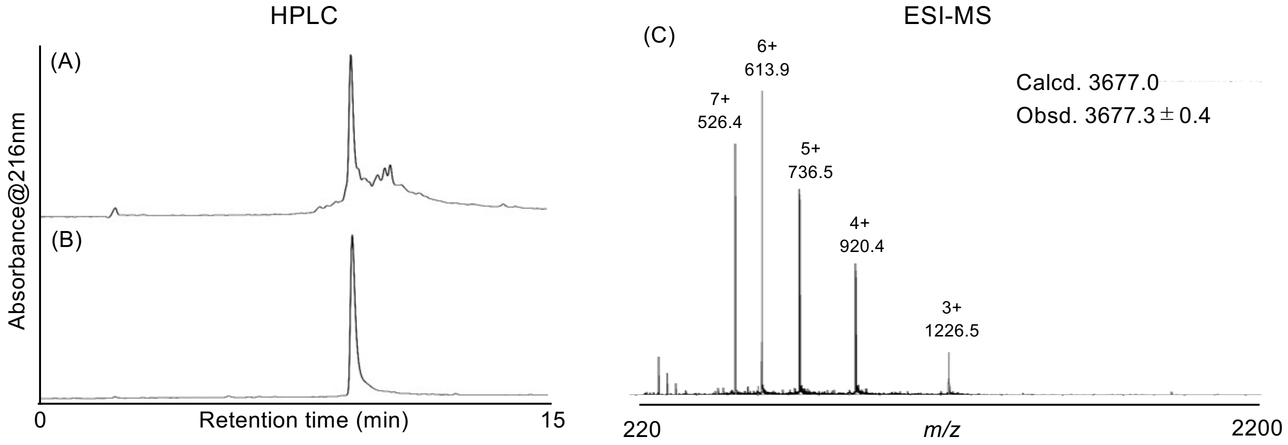

Supplementary Figure S5. Analytical HPLC chromatograms of Ub ($46C^{Acm}$–$48K^{Sez}$–76)-α-hydrazide **15**. **(A)** crude peptide after global deprotection and cleavage. **(B)** purified **15**. **(C)** ESI-MS spectrum of purified **15**.

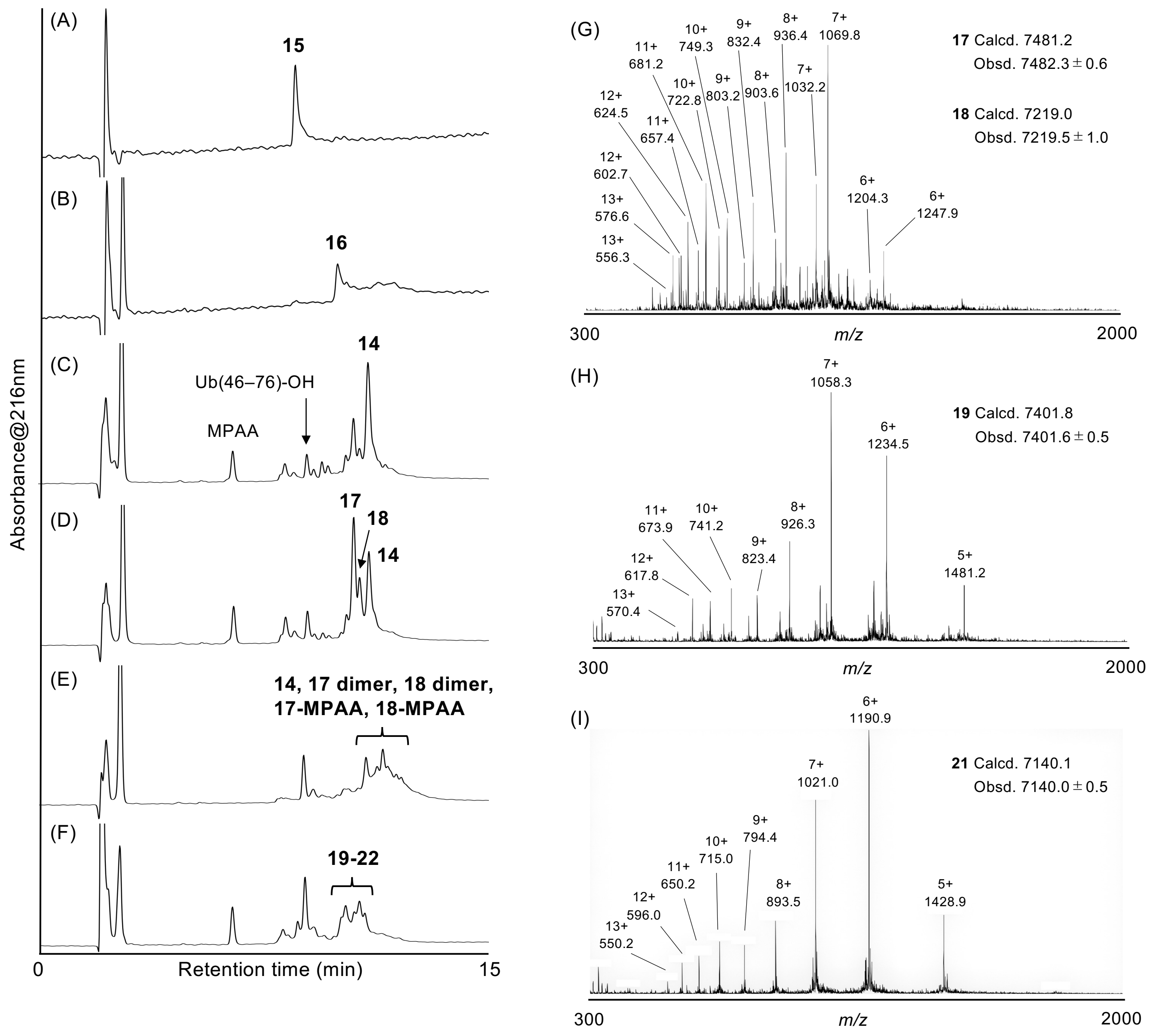

Supplementary Figure S6. Time course of analytical HPLC chromatograms of the first attempt of one-pot δ-selenolysine-mediated isopeptide bond formation. **(A)** Selenazolidine deprotection at 1 min. **(B)** Selenazolidine deprotection at 4.5 h. **(C)** Ligation at 20 min. **(D)** Ligation at 1.5 h. **(E)** Ligation at 13 h. **(F)** Deselenization at 16 h. ESI-MS spectra of **(G) 17** and **18** from (D), **(H) 19** from (F), **(I) 21** from (F).

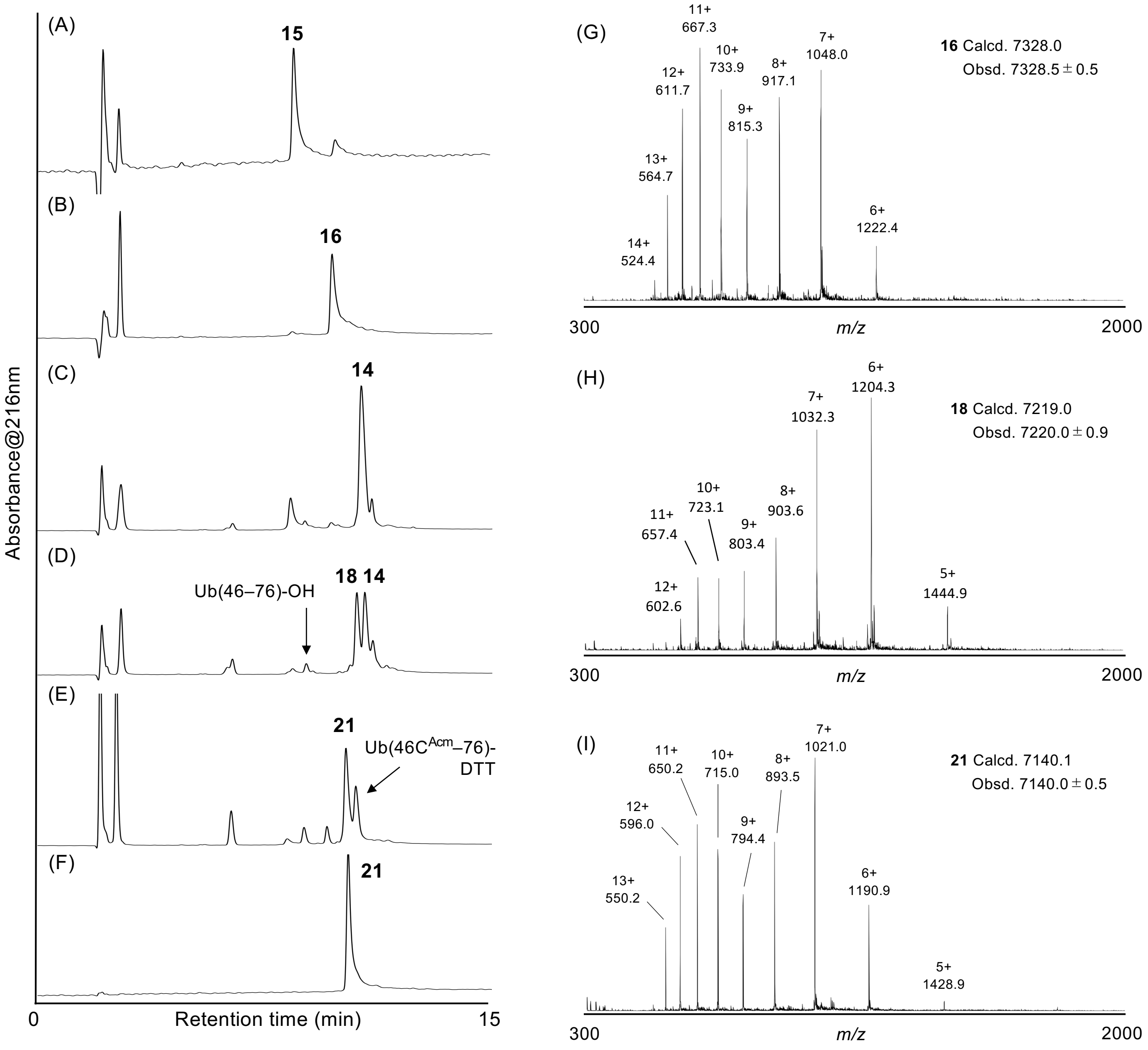

Supplementary Figure S7. Time course of analytical HPLC chromatograms of the one-pot δ-selenolysine-mediated isopeptide bond formation. **(A)** Selenazolidine deprotection at 1 min. **(B)** Selenazolidine deprotection at 30 min. **(C)** Ligation at 1 min. **(D)** Ligation at 2 h. **(E)** Deselenization at 13 h. Peak eluted after **21** is Ub(46C^{Acm}–76)-DTT produced from the remaining **14**. **(F)** Purified **21**. ESI-MS spectra of **(G) 16** from (B), **(H) 18** from (D), **(I)** purified **21**.

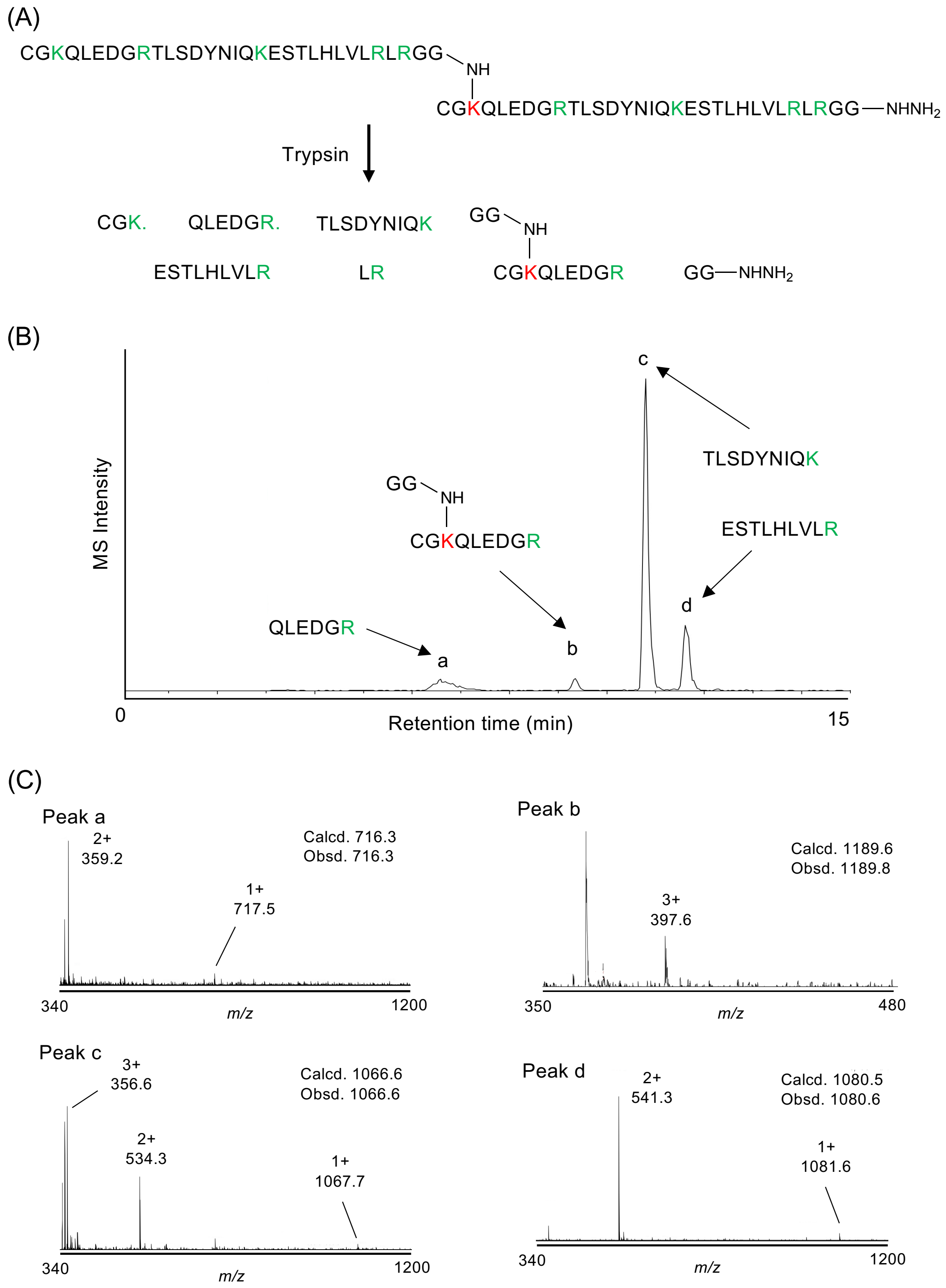

Supplementary Figure S8. (A) Sequence of isopeptide **21** and expected peptide fragments after trypsin digestion. **(B)** LC/MS extracted ion chromatogram of trypsin digestion reaction. *m/z* 359.2:QLDGER, 397.6:CGK(GG)QLEDGR, 534.7:TLSDYNIQK, 534.3:ESTLHLVLR. **(C)** ESI-MS spectra of peaks a, b, c, and d in (B).

4 HRMS, ^{1}H and ^{13}C NMR spectra

HR-FTMS, ^{1}H and ^{13}C NMR spectra of 2

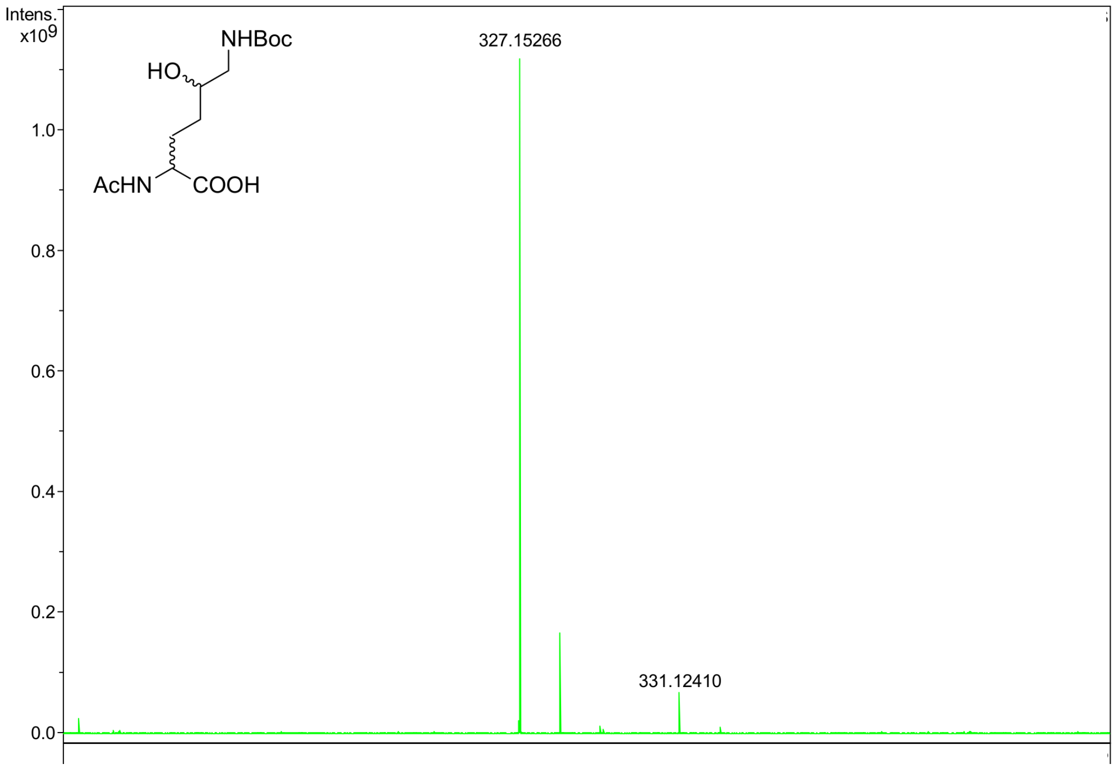

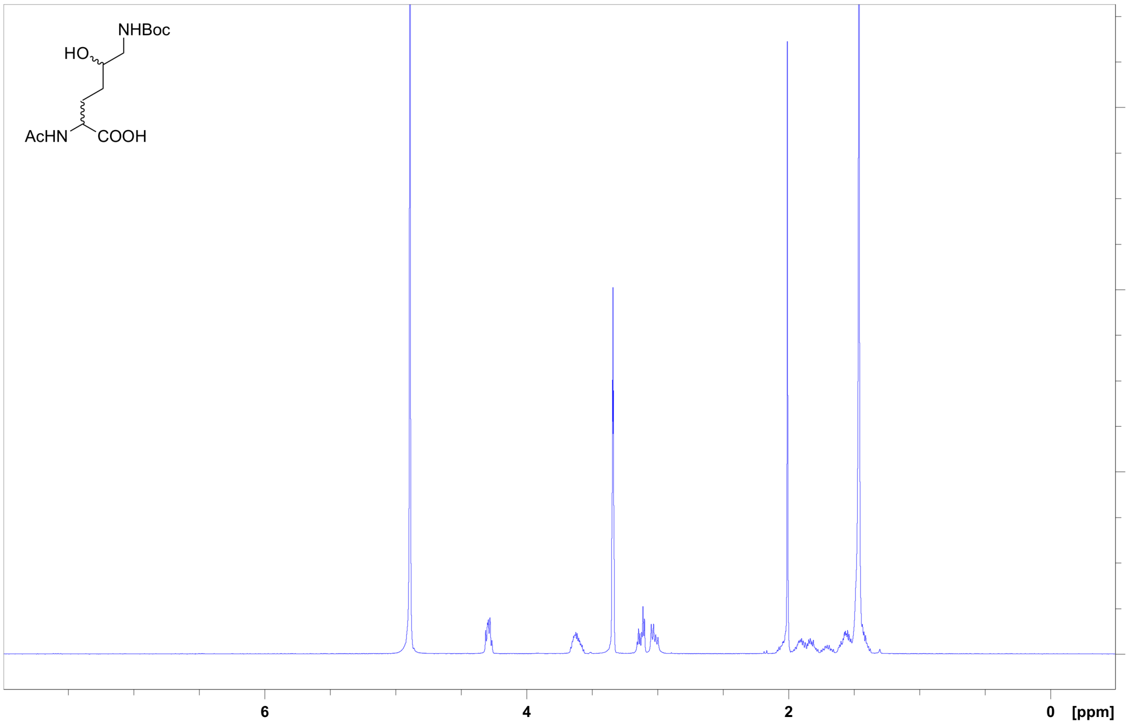

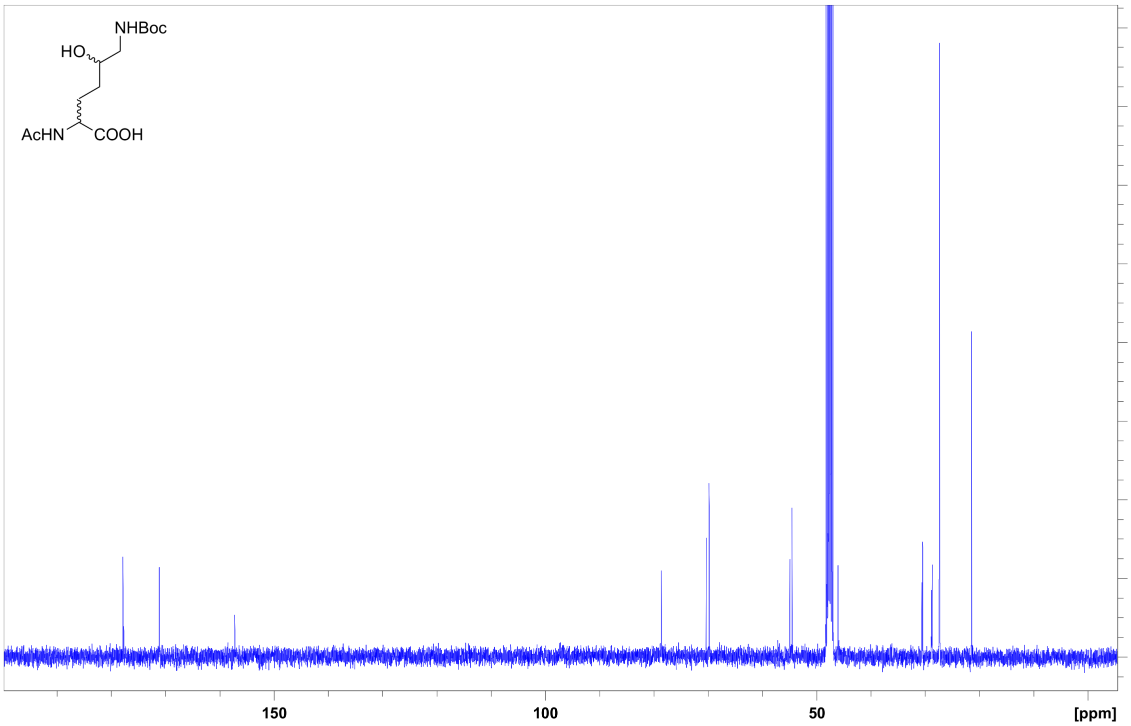
NHBoc
HO
AcHN
COOH
150
100
50
[ppm]

HR-FTMS, ^{1}H and ^{13}C NMR spectra of 3.

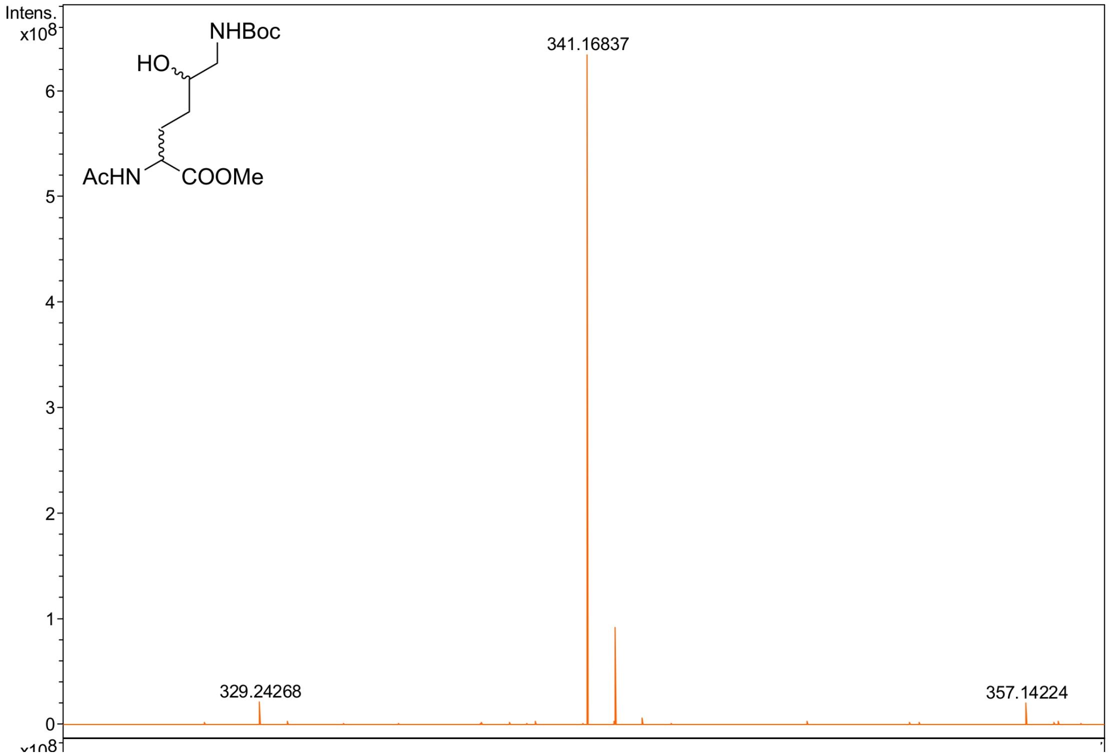

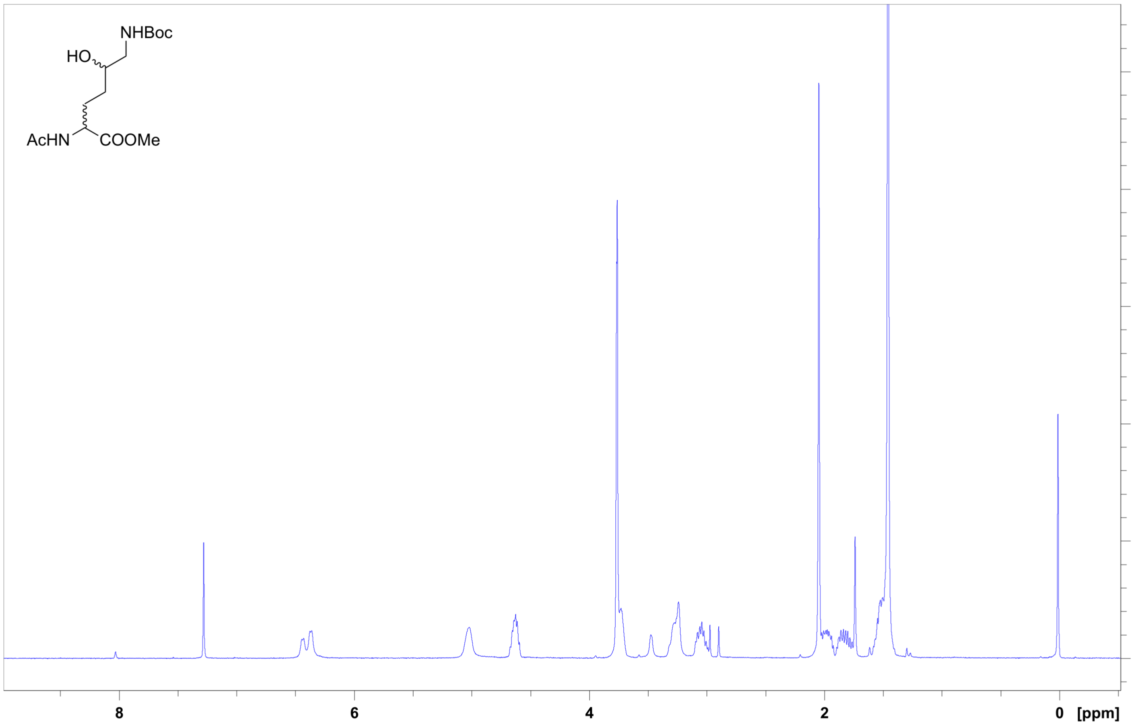

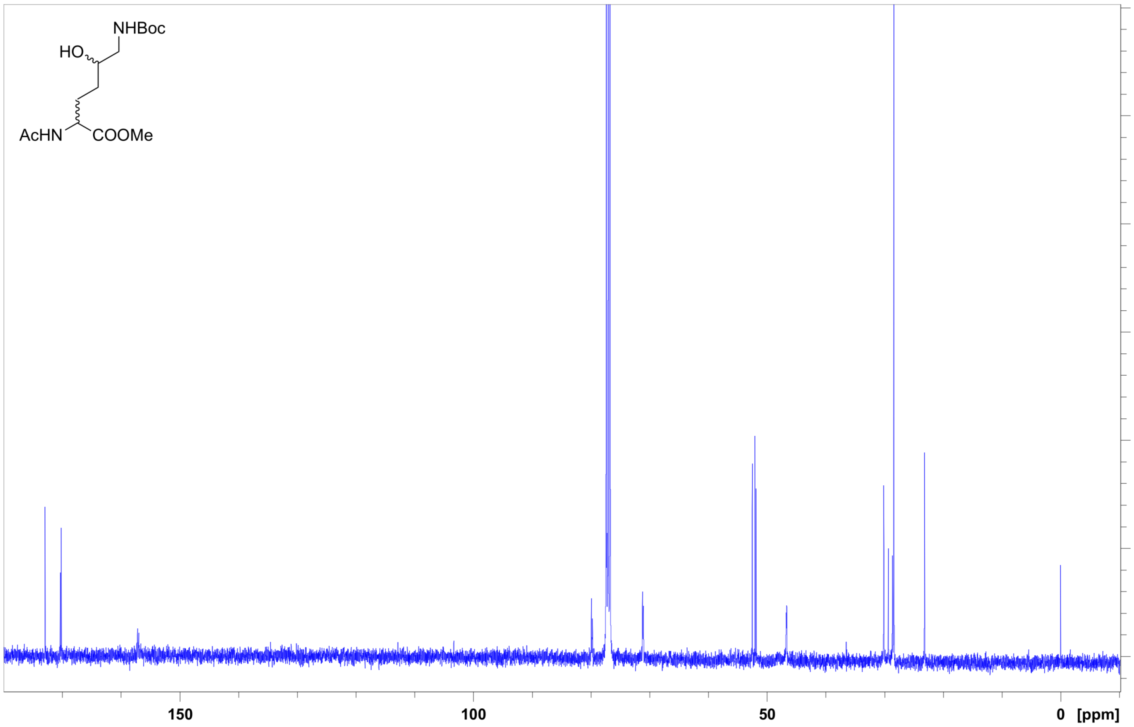
NHBoc
HO
AcHN
COOMe
150
100
50
0
[ppm]

^{1}H and ^{13}C NMR spectra of 4

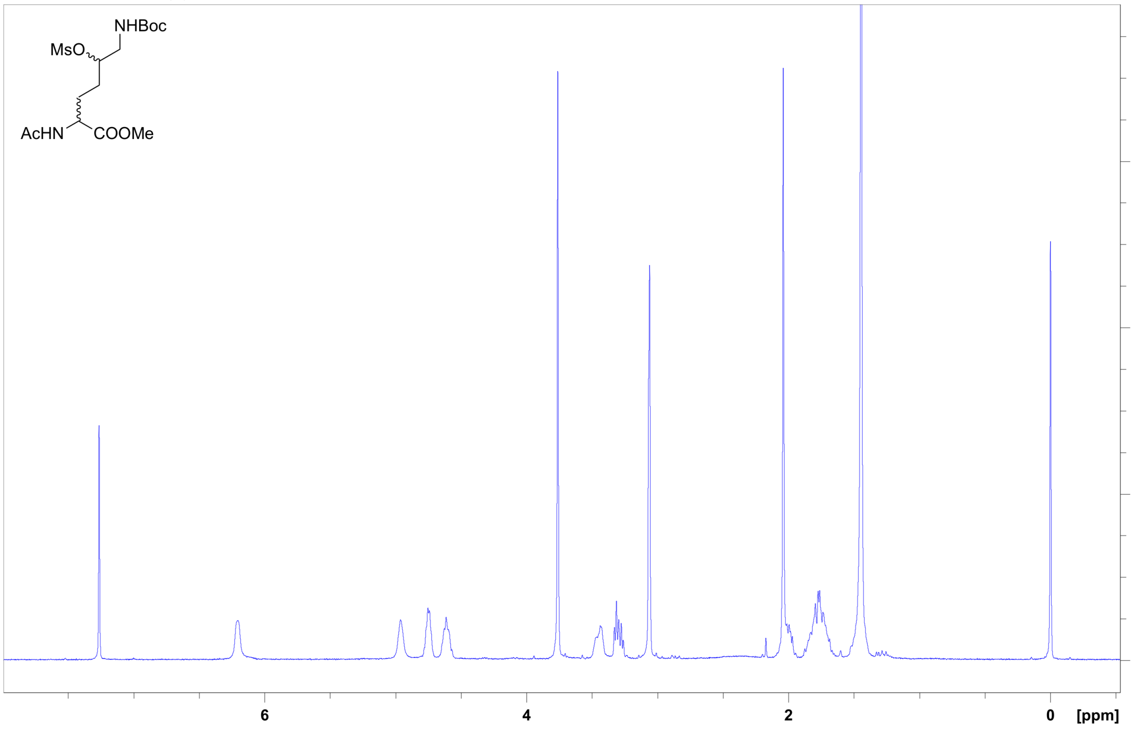

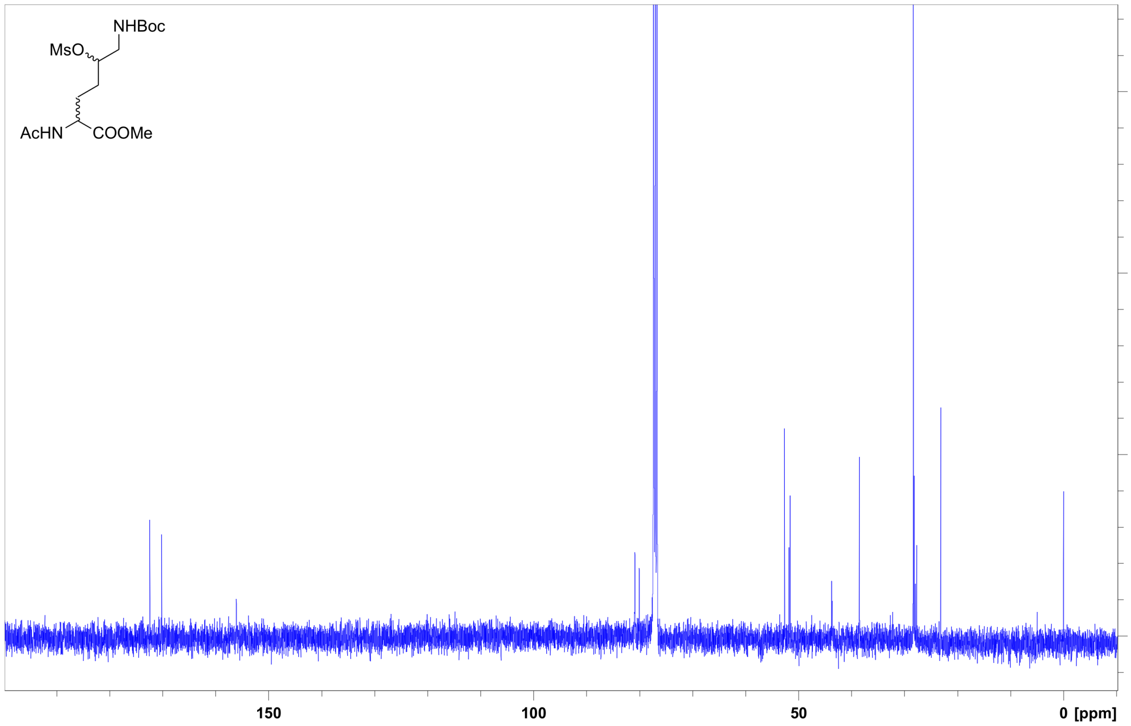

HR-TOFMS, ^{1}H and ^{13}C NMR spectra of 5

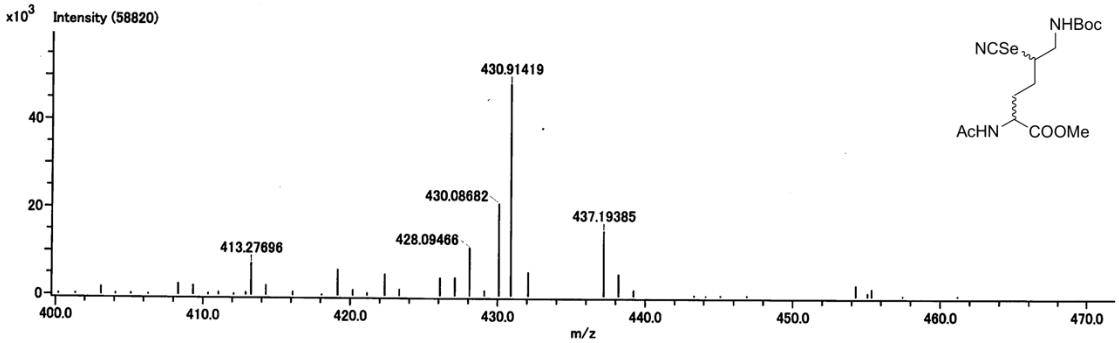

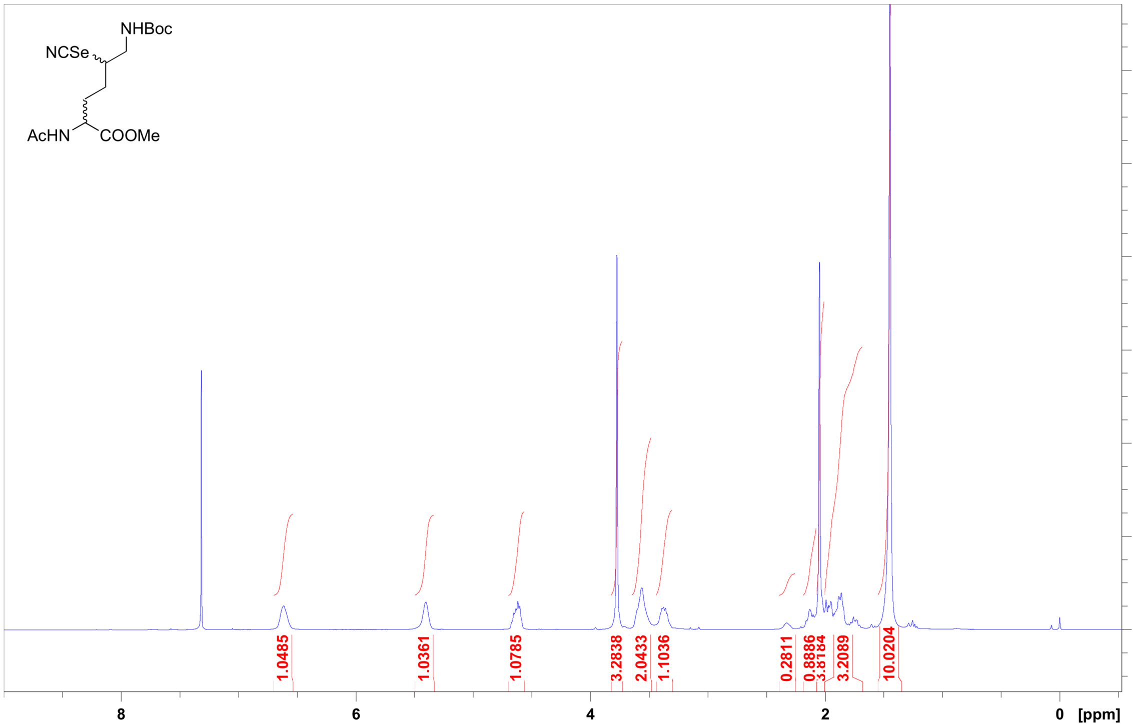

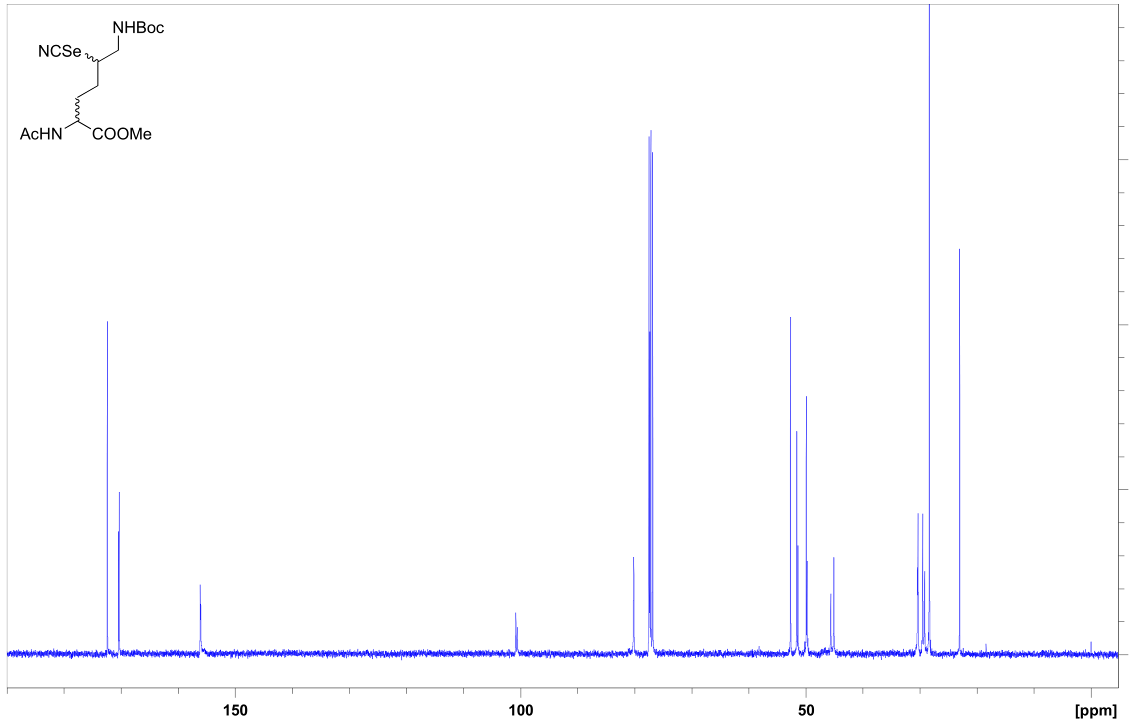
NHBoc
NCSe
AcHN
COOMe
150
100
50
[ppm]

^{1}H and ^{13}C NMR spectra of 6

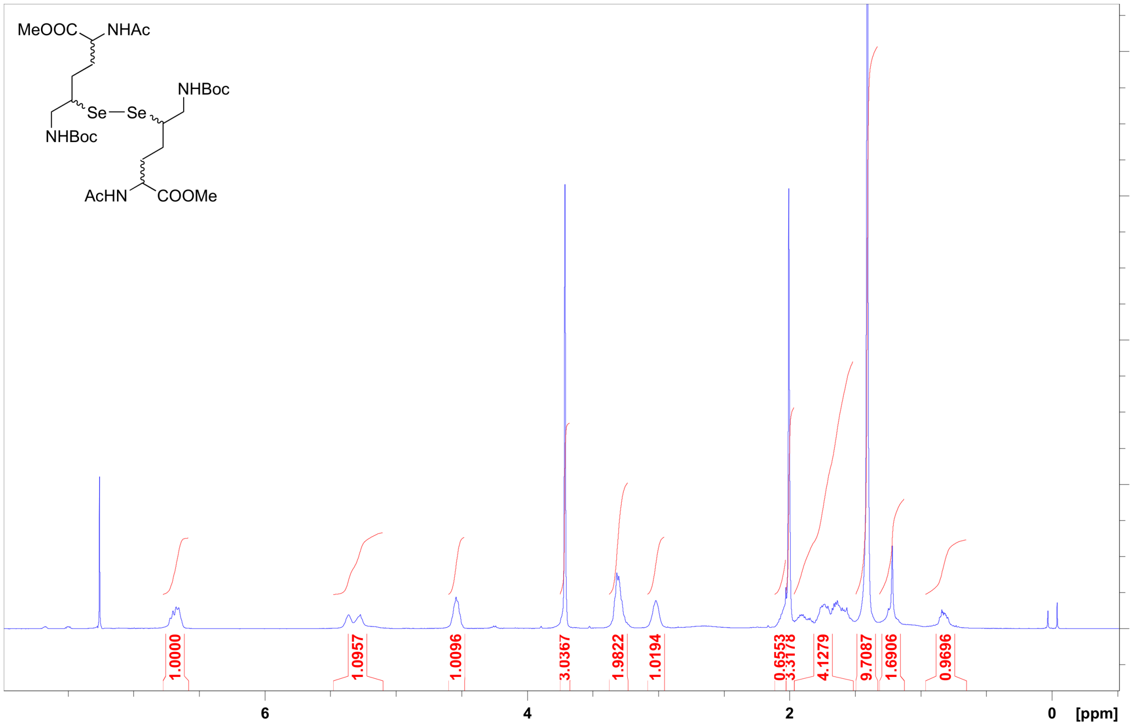

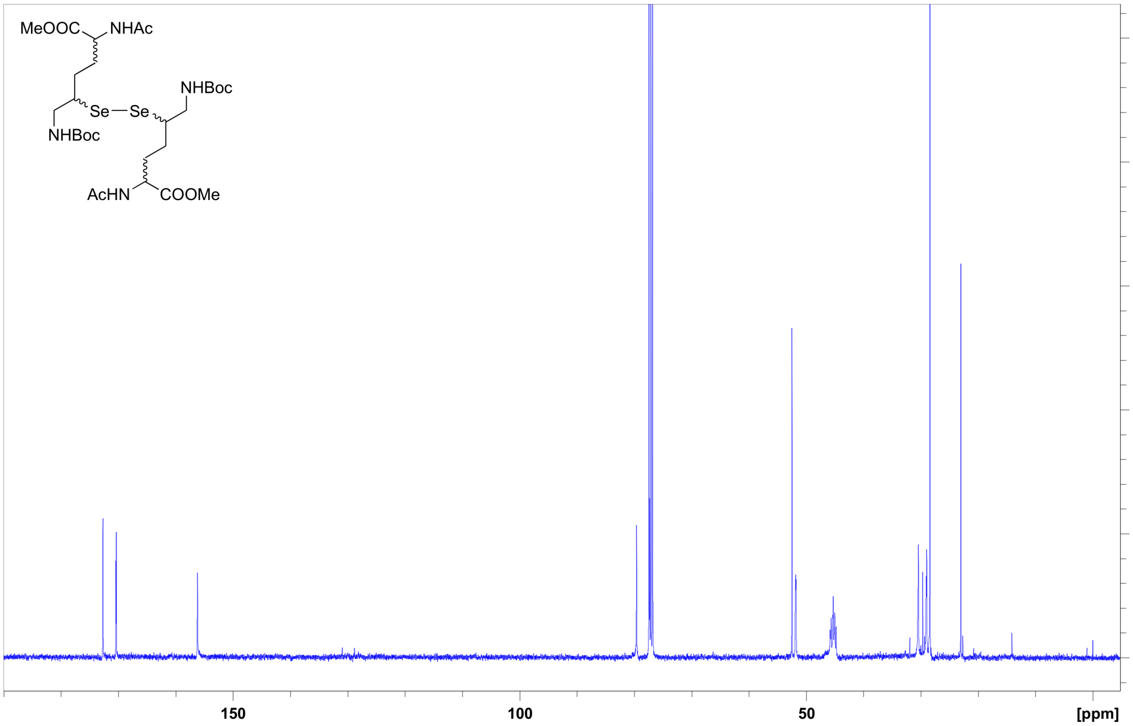

^{1}H and ^{13}C NMR spectra of *N*-α-acetyl-*N*-ε-(*tert*-butoxycarbonyl)-δ,ε-*Se,N*-methylene-δ-DL-seleno-DL-lysine methyl ester

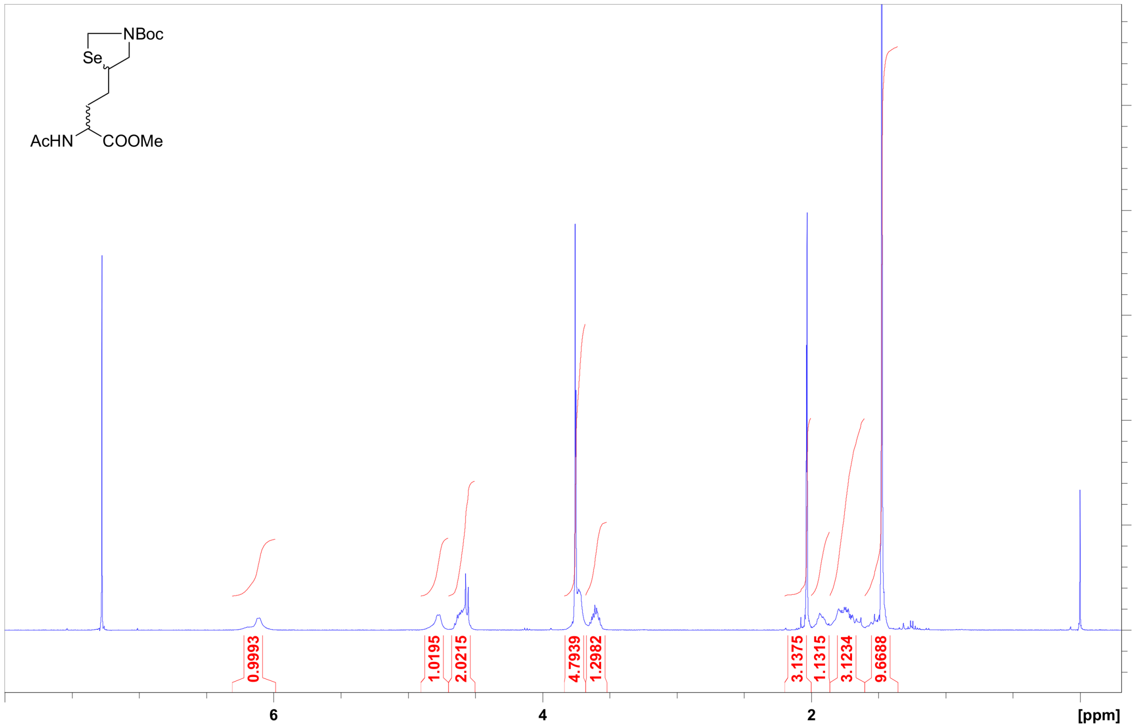

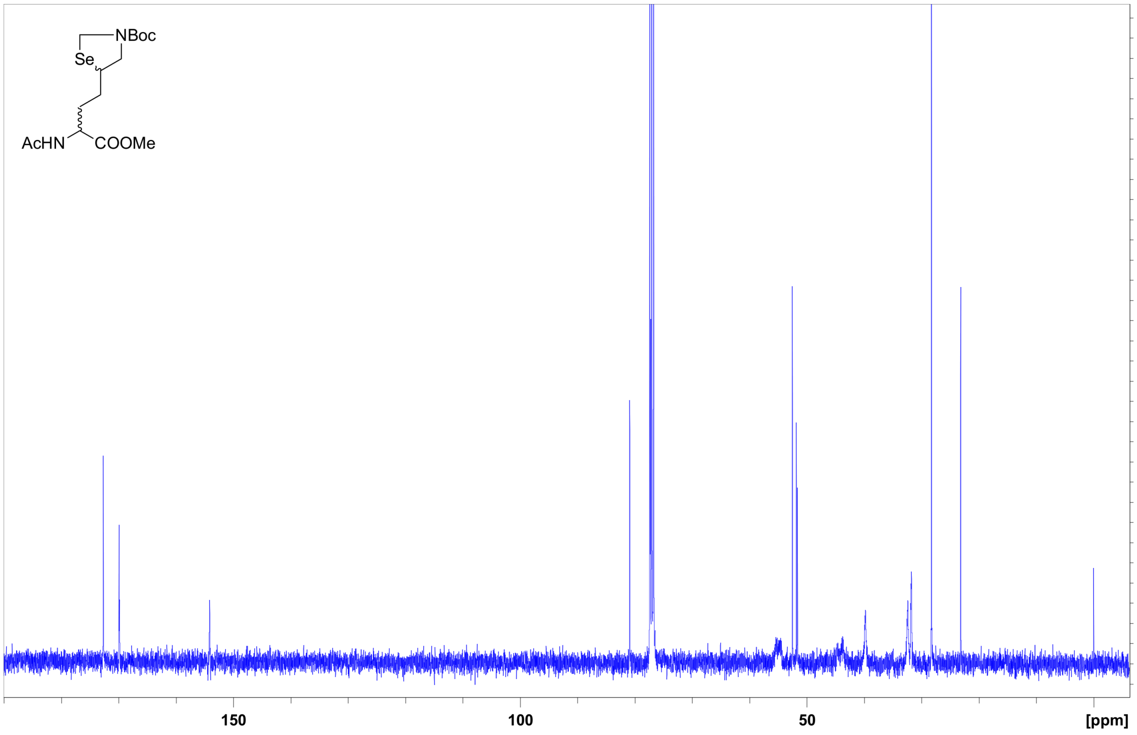

HR-TOFMS, and ^{1}H NMR spectra of 7

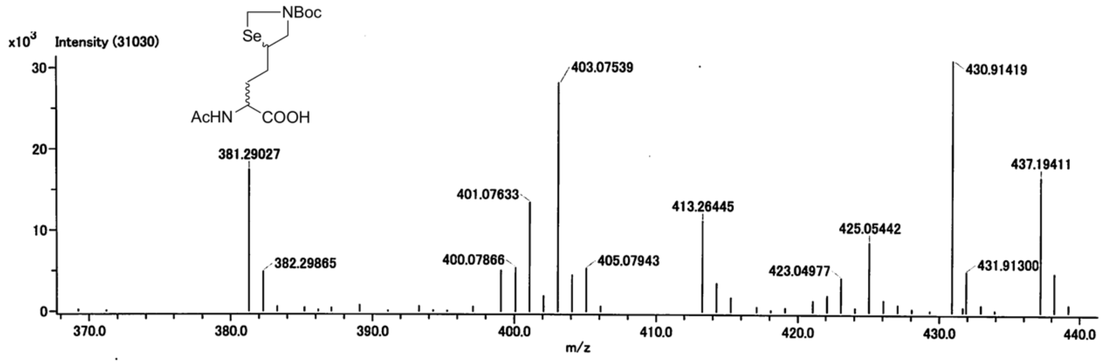

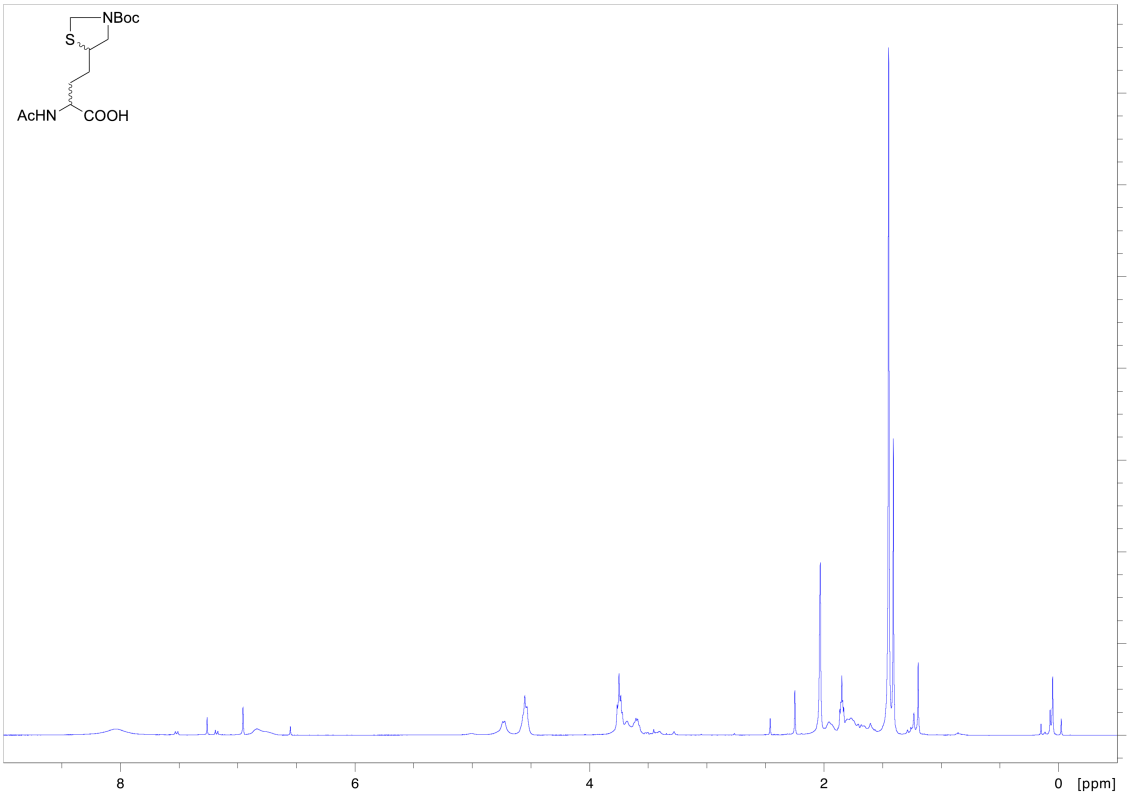

HR-FTMS, ^{1}H, ^{13}C NMR, COSY, and HSQC spectra of L-9

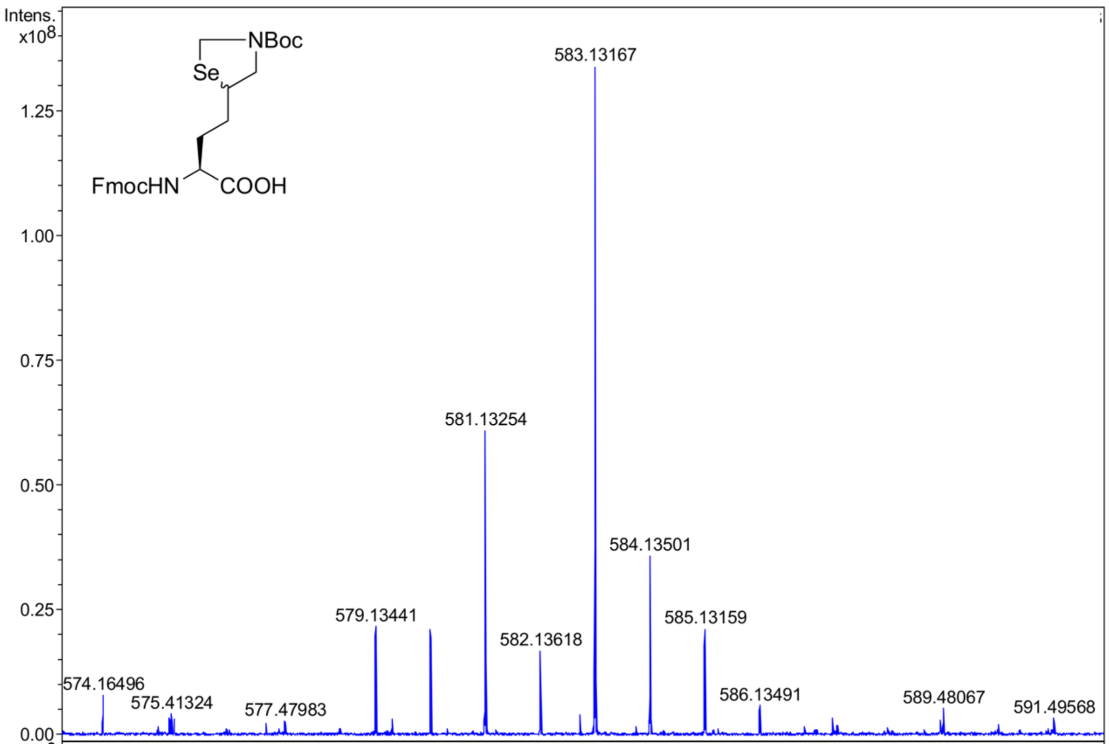

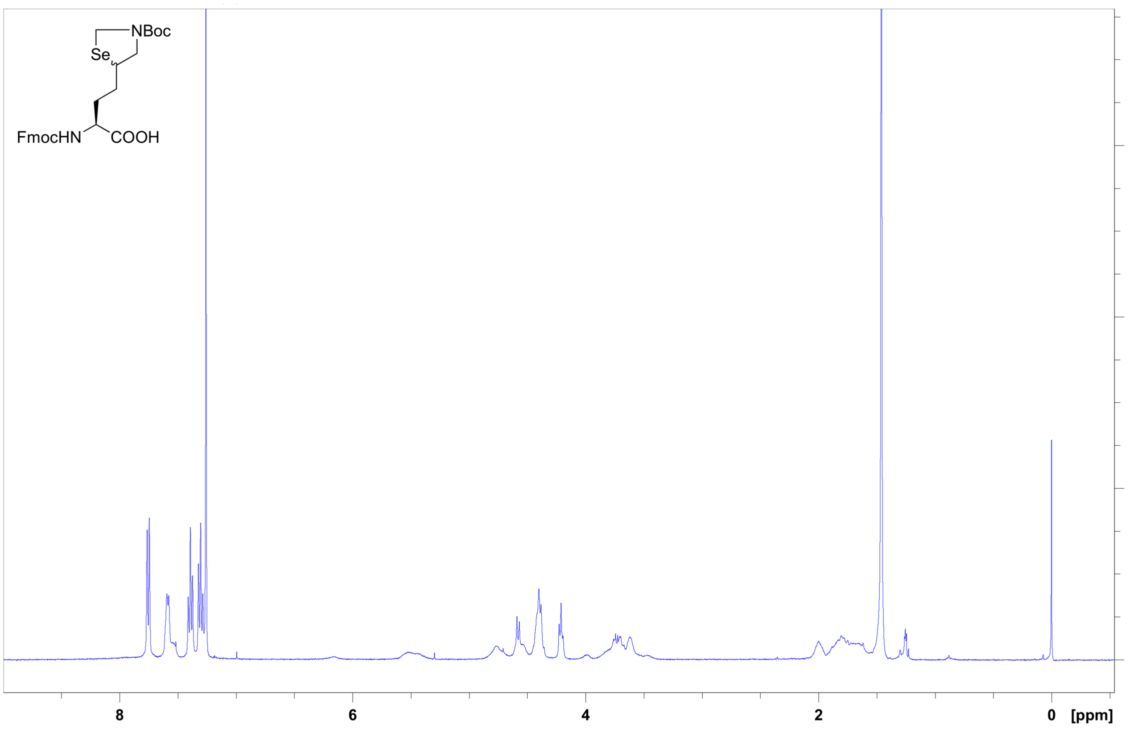
NBoc
Se
FmocHN
COOH
8
6
4
2
0
[ppm]

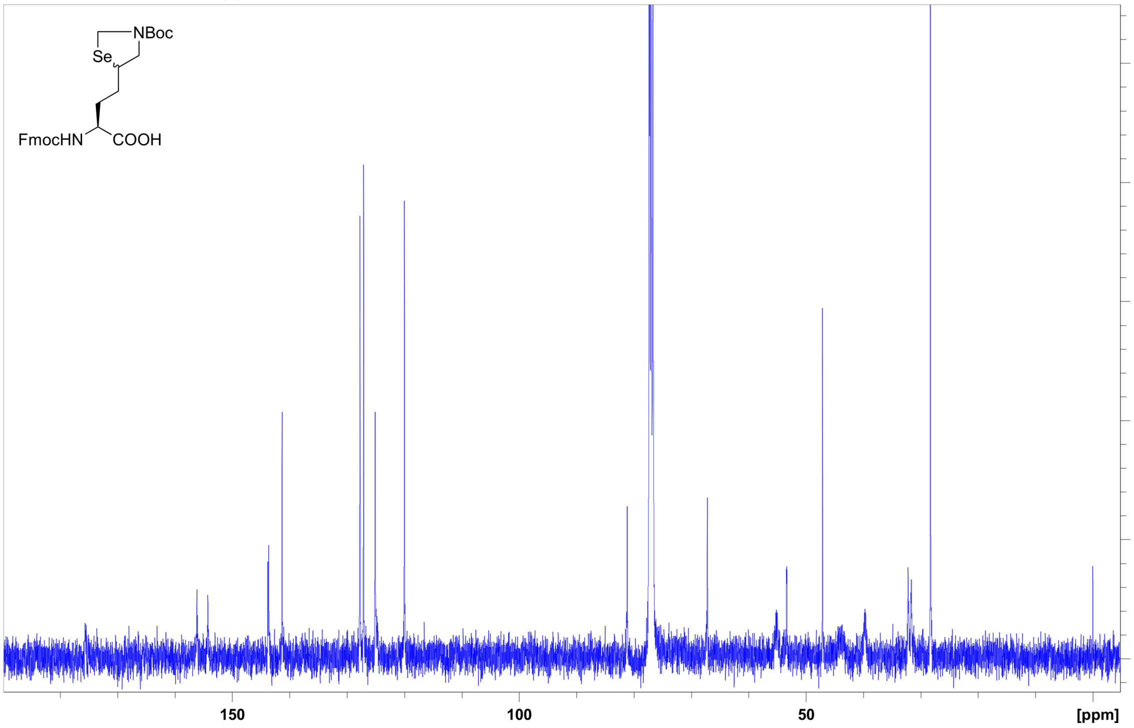
NBoc
Se
FmocHN
COOH
150
100
50
[ppm]

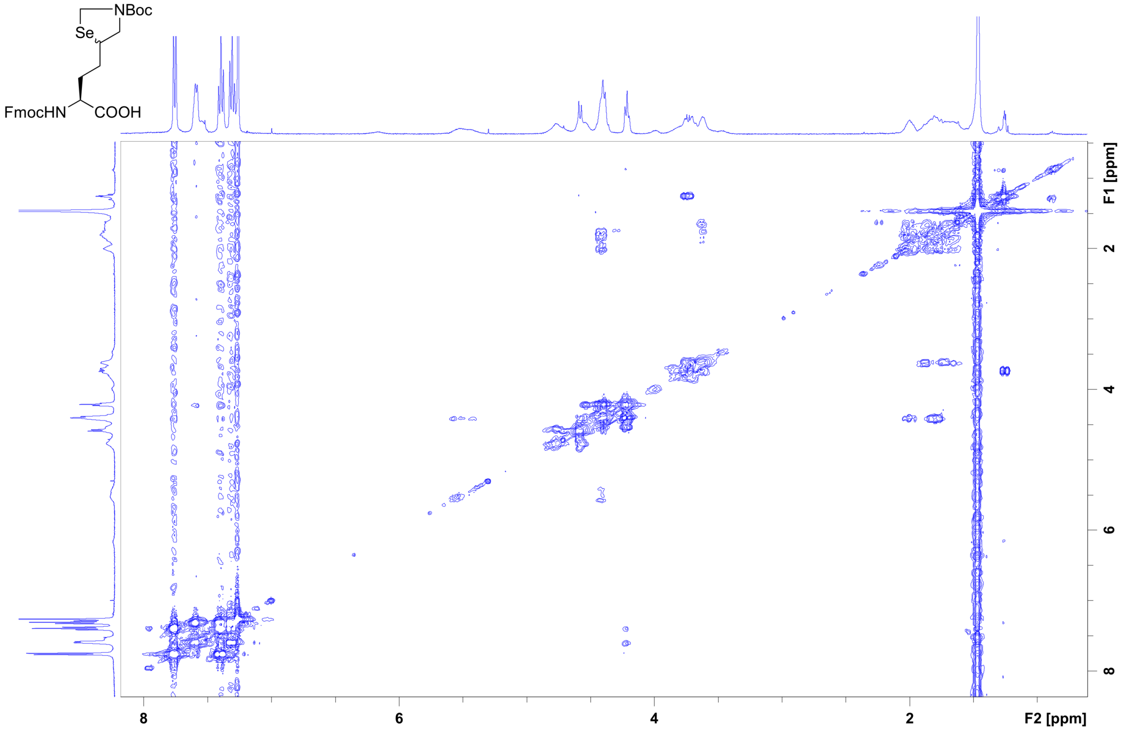
NBoc
Se
FmocHN
COOH
F1 [ppm]
F2 [ppm]

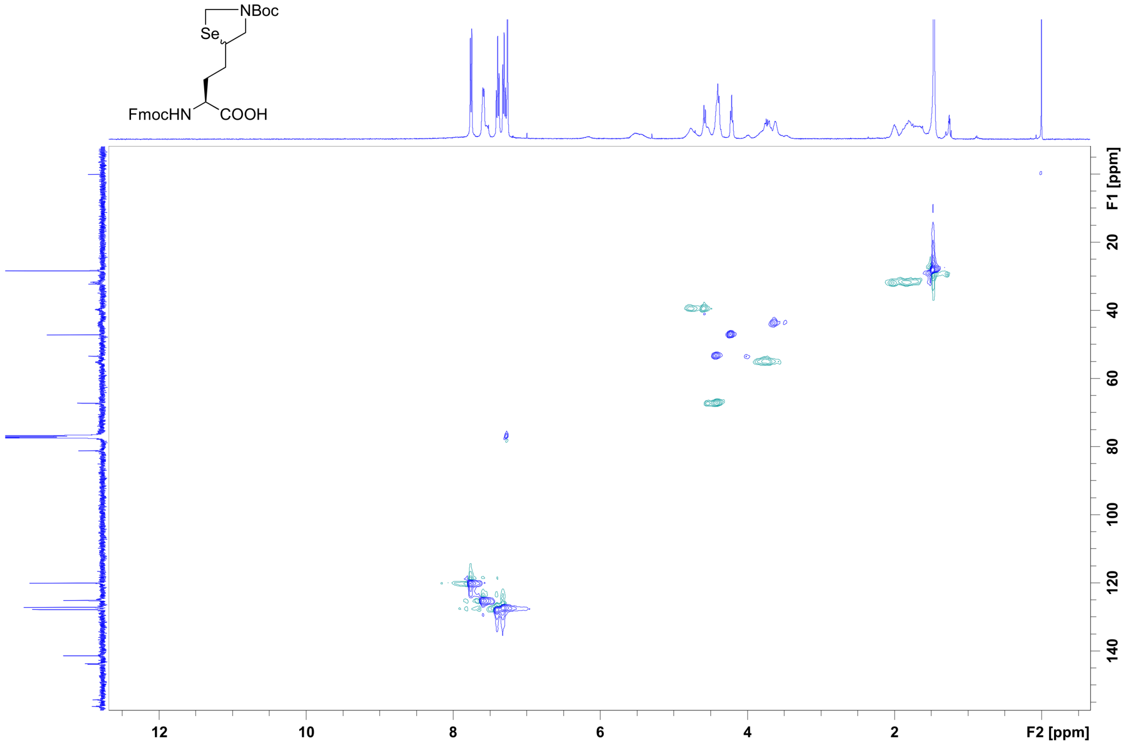
NBoc
Se
FmocHN
COOH
F1 [ppm]
F2 [ppm]

^{1}H NMR spectrum of D-9

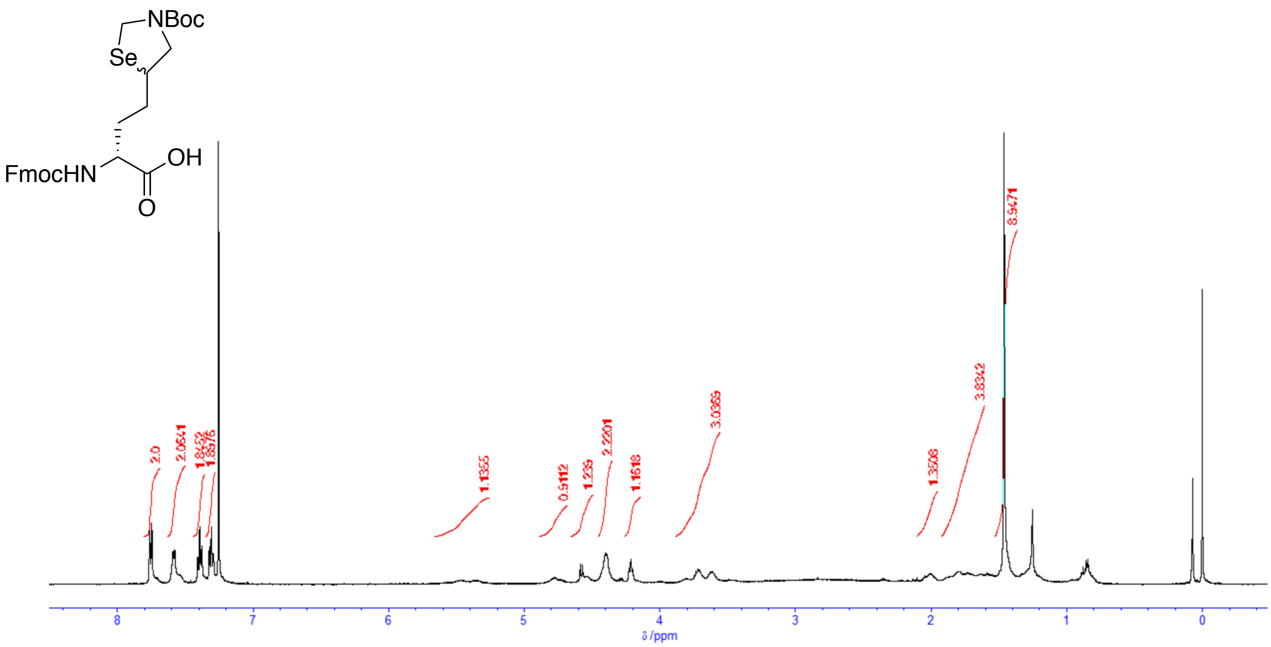

Supplement: Supplementary file 1 [file DataSheet1.pdf]
